# Application of a JA-Ile Biosynthesis Inhibitor to Methyl Jasmonate-Treated Strawberry Fruit Induces Upregulation of Specific MBW Complex-Related Genes and Accumulation of Proanthocyanidins

**DOI:** 10.3390/molecules23061433

**Published:** 2018-06-13

**Authors:** Laura D. Delgado, Paz E. Zúñiga, Nicolás E. Figueroa, Edgar Pastene, Hugo F. Escobar-Sepúlveda, Pablo M. Figueroa, Adrián Garrido-Bigotes, Carlos R. Figueroa

**Affiliations:** 1 Phytohormone Research Laboratory, Institute of Biological Sciences, Universidad de Talca, Talca 3465548, Chile; laudelgadosoto@gmail.com (L.D.D.); paz.biotech@gmail.com (P.E.Z.); ibv.nicfigueroa@gmail.com (N.E.F.); hescobar.sepulveda@gmail.com (H.F.E.-S.); pabfigueroa@utalca.cl (P.M.F.); adriangarrido9@gmail.com (A.G.-B.); 2 Laboratorio de Farmacognosia, Faculty of Pharmacy, Universidad de Concepción, Concepción 4070386, Chile; epastene@udec.cl; 3 Faculty of Forest Sciences, Universidad de Concepción, Concepción 4070386, Chile

**Keywords:** anthocyanins, *Fragaria* × *ananassa*, jasmonates, jarin-1, *MYB* genes

## Abstract

Fleshy fruits are an important source of anthocyanins and proanthocyanidins (PAs), which protect plants against stress, and their consumption provides beneficial effects for human health. In strawberry fruit, the application of exogenous methyl jasmonate (MeJA) upregulates anthocyanin accumulation, although the relationship between the jasmonate pathway and anthocyanin and PA biosynthesis in fruits remains to be understood. Anthocyanin and PA accumulation is mainly regulated at the transcriptional level through R2R3-MYB and bHLH transcription factors in different plant species and organs. Here, the effect of jarin-1, a specific inhibitor of bioactive JA (jasmonoyl-isoleucine, JA-Ile) biosynthesis, on anthocyanin and PA accumulation was evaluated during strawberry (*Fragaria* × *ananassa*) fruit development using an in vitro ripening system for 48 h. Also, we observed the effects of MeJA and the application of jarin-1 to MeJA-treated fruits (MeJA + jarin-1 treatment). We assessed changes of expression levels for the JA-Ile and MeJA biosynthetic (*FaJAR1.2* and *FaJMT*), JA signaling-related (*FaMYC2* and *FaJAZ1*), MYB-bHLH-WD40 (MBW) complex-related (*FabHLH3/33*, *FaMYB9/10/11*, and repressor *FaMYB1*), and anthocyanin and PA biosynthetic *(FaANS*, *FaUFGT*, *FaANR*, and *FaLAR*) genes. In addition, the promoter region of MBW complex-related *MYB* genes was isolated and sequenced. We found a higher redness of strawberry fruit skin and anthocyanin content in MeJA-treated fruits with respect to jarin-1-treated ones concomitant with an upregulation of *FaANS* and *FaUFGT* genes. Inversely, the PA content was higher in jarin-1- and MeJA + jarin-1-treated than in MeJA-treated fruits. MeJA + jarin-1 treatment resulted in an upregulation of *FaANR* and associated transcription factors such as *FabHLH33* and *FaMYB9/11* along with *FaJMT* and *FaJAR1.2*. Finally, we found JA-responsive elements in the promoter regions of *FaMYB1/9/10/11* genes. It is proposed that PA biosynthesis-related genes can be upregulated by the application of jarin-1 to MeJA-treated fruit, thus increasing PA accumulation in strawberry.

## 1. Introduction

Plant polyphenols play a central role in plant fitness, since these compounds are important in plant environment crosstalk, playing a role in plant responses to biotic and abiotic stress, and in flowers and fruits they are important for pollen fertility and animal attraction for pollination and seed dispersion [1,2,3,4]. Moreover, polyphenols have beneficial properties for human health. In this sense, it has been reported that phenolic compounds found in berry fruits have antioxidant, antimutagenic, and free-radical scavenging activities, and increased consumption of phenolic compounds reduces the risk of cardiovascular diseases and certain types of cancer [5,6,7,8]. In this sense, strawberry (*Fragaria* × *ananassa*) consumption has a positive impact on human health, since this fruit is a relevant source of antioxidant compounds [9,10]. Strawberry fruit contains large amounts of phenolic compounds, such as flavonoids, including anthocyanins, flavonols, flavanols, and proanthocyanidins (PAs), followed by hydrolysable tannins and phenolic acids [10,11]. Strawberry fruit contains variable amounts of anthocyanins and PAs during fruit development and ripening, with opposite trends of accumulation: while PAs accumulate in green stages and decrease toward ripening, the reverse is the case for anthocyanins [12,13,14,15,16]. Compared with anthocyanins, PAs are a minority in ripe strawberry fruit [10,13].

Anthocyanins are widely distributed pigments in the plant kingdom and are responsible for blue, purple, violet, and red coloration in most plants, with their presence more obvious in flowers and fruits [17]. On strawberry fruit, pelargonidin-3-glucoside and pelargonidin-3-*O* malonyl-glucoside make up 80% and 14% of the total anthocyanin content, respectively [13], accumulating at the end of the ripening process. The final steps of biosynthesis of anthocyanin pigments in strawberry involve the enzymes dihydroflavonol 4-reductase (DFR), anthocyanidin synthase (ANS), and uridine diphosphate (UDP) glucose:flavonoid 3-*O*-glucosyl transferase (UFGT) [14,18].

Proanthocyanidins, also called condensed tannins, are polymeric flavan-3-ols [19]. Strawberry fruits accumulate 3′-4′-flavan-3-ols, derived from catechin and epicatechin and their corresponding PAs, mainly by the actions of leucoanthocyanidin reductase (LAR) and anthocyanidin reductase (ANR) [14]. PAs are found on tissues such as fruits, leaves, and stems, where their main function is to provide protection against pathogens, insects, and herbivores [19]. On the other hand, different properties associated with benefits for human health have been reported for PAs, among them antioxidant, antimicrobial, antiallergic, and antihypertensive effects [20].

Many highly conserved transcription factors (TFs) regulate the expression of genes involved in late flavonoid/phenylpropanoid metabolism, including anthocyanin and PA biosynthesis. They are mainly R2R3-MYB TFs, interacting or not with basic helix-loop-helix (bHLH) TFs and/or with proteins containing the conserved WD40 repeats to form the so-called ternary MYB-bHLH-WD40 (MBW) complexes [21]. In strawberry, several MBW partners have been characterized for their role in anthocyanin and PA biosynthesis during fruit development and ripening [15,22,23]. Specifically, for PA biosynthesis in strawberry, four MBW-related proteins that are functional homologs of the Arabidopsis *AtTT2* [24], *AtTT8* [25], and *AtTTG1* [26] were characterized and named *FaMYB9/FaMYB11*, *FabHLH3*, and *FaTTG1*, respectively [15]. Moreover, in apple calluses, overexpression of the strawberry orthologs MYB9 and MYB11 proteins promoted PA accumulation [27]. Finally, FaMYB10 and FaMYB1 have been described as a key positive TF and a repressor for ripening-associated anthocyanin accumulation in *F.* × *ananassa* fruit, respectively [22,23,28,29].

On the other hand, plant hormones regulate fruit development and ripening, and they could be related to the accumulation of interesting bioactive compounds in fruit [30]. Unlike other Rosaceae family plants, the strawberry is considered to be a nonclimacteric fruit because the flesh does not ripen in response to the phytohormone ethylene [30,31]; thus, it plays a secondary role in fruit ripening. Other phytohormones possibly serve as major regulators in nonclimacteric fruit ripening. Abscisic acid (ABA) has been found to play a major role in the induction of nonclimacteric fruit ripening, including in strawberry [30,32]. Moreover, the bioactive jasmonate, jasmonoyl-isoleucine (JA-Ile), could play a role in anthocyanin and PA accumulation. To date, few studies have been conducted to assess the role of jasmonates (JAs) in strawberry fruit ripening, although we recently reported a study showing JA-Ile accumulation at early developmental stages and a subsequent decrease through strawberry fruit ripening [33] concomitant with the PA accumulation pattern [13,14].

**Figure 1:**
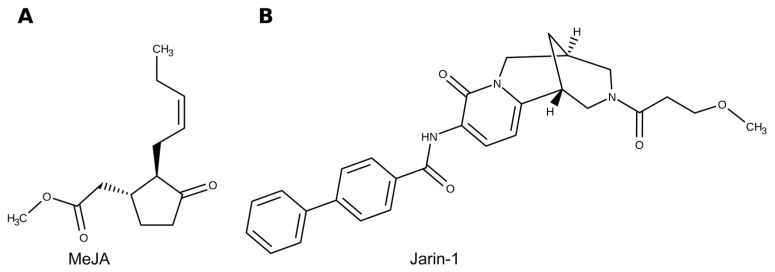
Representation of chemical structures for (**A**) methyl jasmonate (MeJA) and (**B**) jarin-1 molecules used in the present research. Jarin-1 molecule originally reported by Meesters et al. 2014 [41].

In Arabidopsis seedlings, anthocyanin accumulation induced by JAs has been reported [34]. The authors suggest that pigment accumulation may be mediated by an upregulation of MBW-related components, including the MYB-types PAP1 and PAP2 and the bHLH-types GL3 TFs, which could upregulate the expression of genes encoding for DFR and UFGT enzymes that control the last steps of anthocyanin biosynthesis [34]. Regarding the connection between JA signaling and anthocyanin biosynthesis, a bHLH TF, MYC2, has been shown to be a positive regulator of JA-mediated flavonoid biosynthesis in Arabidopsis, along with the other protein family members MYC3 and MYC4 [35,36]. Moreover, previous studies showed that exogenous application of methyl jasmonate (MeJA; Figure 1A) on strawberry fruits accelerated red color acquisition, together with an improvement of other fruit quality attributes, through greater and transient anthocyanin accumulation [37,38,39,40]. The anthocyanin accumulation in MeJA-treated Chilean strawberry (*Fragaria chiloensis*) fruit is related to an upregulation of the corresponding biosynthetic genes, among them *DFR*, *ANS*, and *UFGT* [38]. Finally, it has been described that along with anthocyanin accumulation and color acquisition, MeJA application to developing strawberry fruits induces the accumulation of JA-Ile [33].

Additionally, jarin-1 (from jasmonic acid:amino acid synthetase (JAR1) inhibitor; Figure 1B) was validated as a chemical inhibitor able to prevent jasmonic acid (JA) conversion into JA-Ile mediated by JAR1 in Arabidopsis [41]. Through molecular, biochemical, and chemical approaches, the enzyme JAR1 was identified as the molecular target of jarin-1 [41]. In this study, a decrease in anthocyanin accumulation and JAR1 activity was also reported for jarin-1-treated plants. In this sense, jarin-1 is an effective and promising tool for further studies on JA-Ile-related responses in Arabidopsis and other species.

Recently, our group identified and characterized the key JA metabolism- and signaling-related molecular components in strawberry at the genetic and transcriptional levels [16,33]. Specifically, we reported a downregulated transcriptional profile of the encoding genes for JA-Ile and MeJA biosynthesis-related enzymes JAR1 and jasmonic acid methyl transferase (JMT), respectively [33], and for the key signaling components MYC2 transcription factor and jasmonate ZIM-domain repressors (JAZs) [16] from early developmental to ripe fruit stages. However, it is worth noting that the role of JA-Ile in anthocyanin and PA accumulation in nonclimacteric fruit has not been fully elucidated; thus, the effect of chemicals that affect endogenous JA levels, and in this manner the anthocyanin and PA contents could be assessed. Consequently, the present work evaluates the effect of jarin-1 (alone and applied to MeJA-treated fruit) on the anthocyanin and PA contents in strawberry (*Fragaria* × *ananassa*) fruits, and on transcriptional levels of genes encoding for MBW complex-associated TF and JA biosynthesis-related enzymes. We show that jarin-1 application to MeJA-treated fruit upregulates genes encoding for key MYB and bHLH components and increases PA level.

## 2. Results

### 2.1. Effects on Fruit Skin Color, Firmness, and Weight

**Scheme 1:**
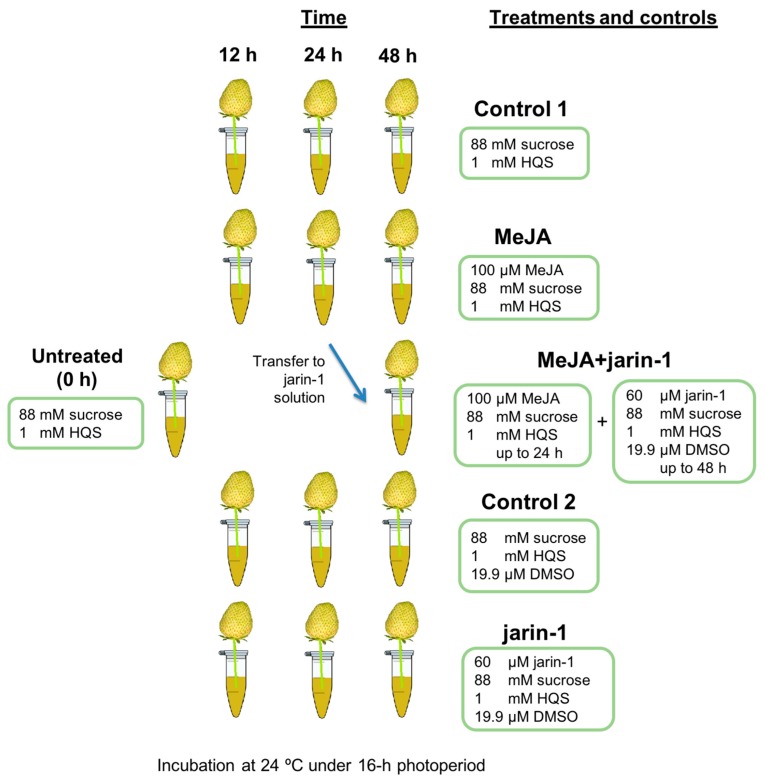
Representation of the experimental design, treatments, and sampling times used in the present research. Five treatments were performed to strawberry (*Fragaria* × *ananassa* cv. Albion) fruits at large green developmental stage arranged in an in vitro ripening system [38,42] for 12, 24, and 48 h maintained at 24 °C under a 16 h photoperiod. Treatment solutions were 100 µM methyl jasmonate (MeJA treatment), 60 µM jarin-1 (jarin-1 treatment), 100 µM MeJA up to 24 h and 60 µM jarin-1 up to 48 h (MeJA + jarin-1 treatment), and the corresponding controls (Control 1 and 2). Six fruits were used for each time point per treatment for different analysis. For experimental details, see the Materials and Methods section. HQS, hydroxyquinoline hemisulfate; DMSO, dimethyl sulfoxide.

First of all, as a main research objective, we proposed to analyze the effects of a JA-Ile biosynthesis inhibitor, jarin-1, on anthocyanin and PA accumulation during strawberry fruit ripening. Thus, we planned an experiment in which the expected MeJA-induced effects could be counteracted by jarin-1 in an in vitro fruit ripening system [38,42]. Therefore, jarin-1 was applied to 24 h MeJA-treated fruit and the effects were observed at the next 24 h (48 h total time treatment). The experimental design, treatments, and sampling times are shown in Scheme 1.

**Figure 2:**
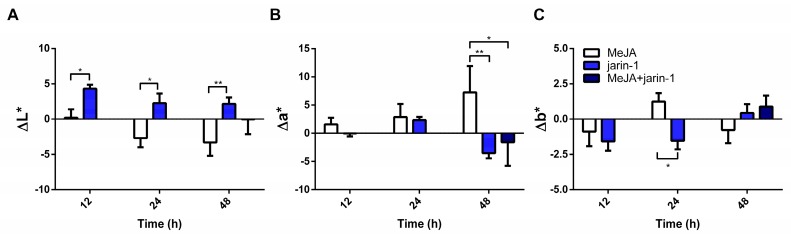
Changes in fruit skin color according to the CIELAB scale ((**A**) L*, (**B**) a*, (**C**) b*) at different time points under treatment compared to respective controls during the in vitro ripening of strawberry fruits. L*, a*, and b* indicate lightness, chromaticity on a green (−) to red (+) axis, and on a blue (−) to yellow (+) axis, respectively. The values were normalized against controls and correspond to the mean of three biological replicates ± S.E. Differences between means were determined using two-way ANOVA and Tukey test. Asterisks indicate significant differences (* *p* < 0.05, ** *p* < 0.01).

Gain or loss in fruit color values could reflect changes in anthocyanin and PA contents. Accordingly, to record changes in fruit skin color, we measured L*, a*, and b* along with color dimensions chroma and hue angle (h°) (Figure 2, Supplementary Table S1). Jarin-1-treated fruit showed higher ΔL* values compared with MeJA at each treatment time (Figure 2A), which could indicate an increase in fruit lightness. On the other hand, MeJA-treated fruits showed a constant increment in Δa* during treatment, reaching the highest Δa* and lowest Δh° values at 48 h compared with jarin-1 and MeJA + jarin-1 treatments (Figure 2B, Supplementary Table S1), which means a significant increase in the acquisition of red color. It is important to note that the application of jarin-1 to MeJA-treated fruits (MeJA + jarin-1) reduced the gain in a* value at 48 h with respect to MeJA treatment (Figure 2B, Supplementary Table S1). No significant differences between treatments were observed in Δb*, with the exception of a lower value in jarin-1-treated fruit at 24 h in relation to MeJA treatment (Figure 2C, Supplementary Table S1).

Regarding changes in fruit firmness and weight, jarin-1-treated fruit exhibited significant firmness reduction compared with its respective control at 12 and 24 h (Supplementary Table S2). A significant minor decrease in softening was observed in MeJA-treated fruits compared with jarin-1-treated ones at 12 h (Supplementary Table S2). Increments in fruit weight were observed in MeJA and jarin-1 treatments at 12 and 24 h compared with their controls, respectively. MeJA + jarin-1 treatment produced a minor weight gain compared to MeJA treatment (Supplementary Table S2). 

### 2.2. Effects on Anthocyanin and PA Contents

**Figure 3:**
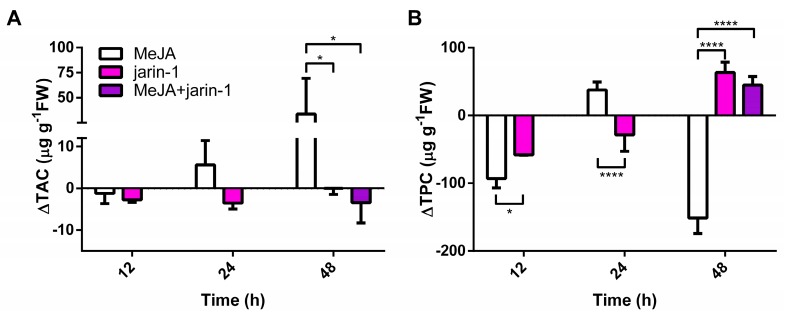
Changes in (**A**) total anthocyanin content (TAC; µg pelargonidin-3-glucoside equivalent per g of fresh weight (FW)) and (**B**) total proanthocyanidin content (TPC; µg catechin equivalent per g of FW) at different time points under treatment compared to respective controls during the in vitro ripening of strawberry fruits. The values were normalized against controls and correspond to the mean of three biological replicates ± S.E. Differences between means were determined using two-way ANOVA and Tukey test. Asterisks indicate significant differences (* *p* < 0.05, **** *p* < 0.0001).

The changes observed in the fruit skin color parameters (Figure 2) suggest that anthocyanin and PA contents could be altered. In this sense, we detected a high increment in total anthocyanin content (TAC) in MeJA-treated fruits at 48 h with respect to the other treatments (Figure 3A, Supplementary Table S3), which supports the higher Δa* and lower Δh° values observed in MeJA-treated fruits at that time (Figure 2B, Supplementary Table S1).

Opposite to the observed results in TAC, an increase and decrease in total proanthocyanidin content (TPC), respectively, were observed in jarin-1- and MeJA-treated fruit at 48 h (Figure 3B, Supplementary Table S4). Jarin-1 and MeJA + jarin-1 treatments showed similar higher values of TPC at 48 h as that observed for MeJA-treated fruit. 

Later, for the purpose of performing a qualitative analysis of PAs, we determined the mean degree of polymerization (mDP) of total PAs and found lower values in jarin-1- and MeJA + jarin-1-treated fruit at 48 h in mDP3 (%) (Supplementary Table S5), which means that trimeric PAs are less represented in these fruit samples.

### 2.3. Effects on Anthocyanin and PA Biosynthesis-Related Gene Expression

#### 2.3.1. Expression Profiles for MBW Complex-Related Genes

**Figure 4:**
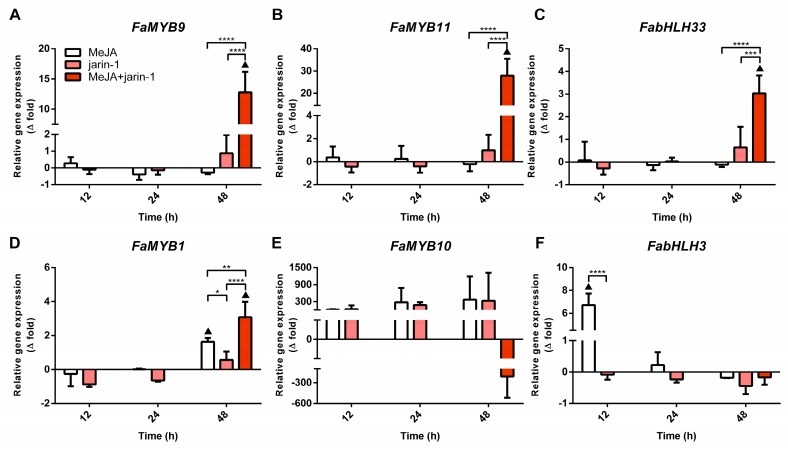
Changes in relative expression levels of (**A**) *FaMYB9*, (**B**) *FaMYB11*, (**C**) *FabHLH33*, (**D**) *FaMYB1*, (**E**) *FaMYB10*, and (**F**) *FabHLH3* at different time points under treatment compared to respective controls during the in vitro ripening of strawberry fruits. The expression data were determined by RT-qPCR and correspond to the mean of three biological replicates ± S.E normalized against controls. Differences between means were determined using two-way ANOVA and Tukey test. Asterisks indicate a significant difference (* *p* < 0.05, ** *p* < 0.01, *** *p* < 0.001, and **** *p* < 0.0001). Triangles (▲) above bars represent significant differences between treatments and their respective controls (*p* < 0.05).

With the aim of gaining insight into the differences in TAC and TPC accumulation observed in the different treatments, we performed an expression analysis for genes encoding for the MBW complex components FabHLH3, FabHLH33, FaMYB9, FaMYB10, and FaMYB11 and the repressor FaMYB1 (Figure 4, Supplementary Table S6). A similar trend was found between the *FaMYB9*, *FaMYB11*, and *FabHLH33* expression patterns, with the highest expression level in MeJA + jarin-1 treatment at 48 h compared to its respective control and other treatments (Figure 4A–C), which could be related to the observed PA accumulation pattern in the same treatment (Figure 3B). 

In turn, *FaMYB1* expression levels presented the highest increase in the MeJA + jarin-1 treatment, although MeJA treatment also increased its level at 48 h (Figure 4D). *FaMYB10* showed constant relative expression levels during all treatment times (Figure 4E). In the case of *FabHLH3*, a significantly higher expression level was observed in MeJA regarding its control and jarin-1 treatment at 12 h, and then no significant differences between treatments were observed (Figure 4F).

**Figure 5:**
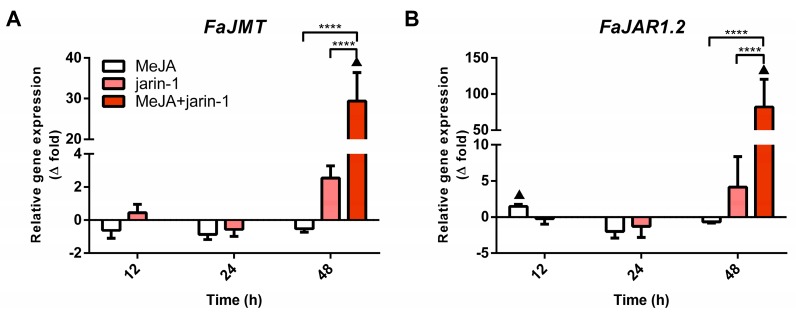
Changes in relative expression levels of (**A**) *FaJMT* and (**B**) *FaJAR1.2* at different time points under treatment compared to respective controls during the in vitro ripening of strawberry fruits. The expression data were determined by RT-qPCR and correspond to the mean of three biological replicates ± S.E normalized against controls. Differences between means were determined using two-way ANOVA and Tukey test. Asterisks indicate significant difference (**** *p* < 0.0001). Triangles (▲) above bars represent significant differences between treatment and respective controls (*p* < 0.05).

Simultaneously, the expression patterns of *FaMYB9*, *FaMYB11*, and *FabHLH33* (Figure 4A–C) were similar to those observed for the encoding genes for MeJA and JA-Ile biosynthesis-related enzymes FaJMT and FaJAR1, respectively (Figure 5, Supplementary Table S7).

Additionally, with the objective of finding some clues to the expression regulation of the *MYB*-related genes, we isolated, sequenced, and performed an in silico analysis of promoter regions for *FaMYB1* (1500 bp; GenBank MH045151), *FaMYB9* (1900 bp; GenBank MH045152), *FaMYB10* (1700 bp; GenBank MH045153), and *FaMYB11* (1700 bp; GenBank MH045154). Several phytohormone-related *cis*-acting elements were identified in a 1500 bp region of the promoters (Supplementary Figure S1, Supplementary Table S8). By using the PlantCARE database, we found ABA-, gibberellin (GA)-, and salicylic acid (SA)-responsive elements in the promoter regions of all *MYB* genes, and ethylene (ET)-responsive elements were observed in *FaMYB9* and *FaMYB11* promoter regions. Specifically, the putative JA-responsive element G-box and its variants were identified in the promoter regions of *FaMYB1* (5′-CACGTGT-3′), *FaMYB9* (5′-AACGTG-3′ and 5′-CACGTT-3′), *FaMYB10* (three 5′-CACGTT-3′), and *FaMYB11* (5′-CACATG-3′).

#### 2.3.2. Expression Profile of Anthocyanin and PA Biosynthesis-Related Genes

**Figure 6:**
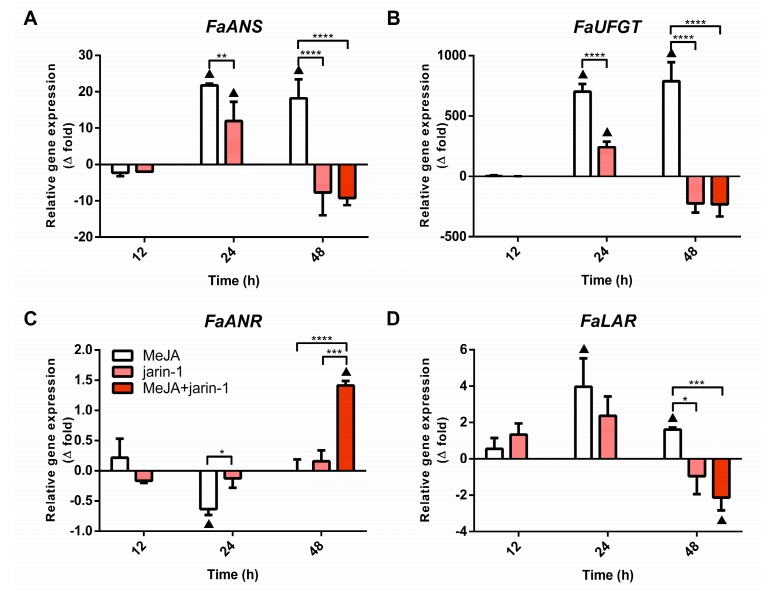
Changes in relative expression levels of (**A**) *FaANS*, (**B**) *FaUFGT*, (**C**) *FaANR*, and (**D**) *FaLAR* at different time points under treatment compared to respective controls during the in vitro ripening of strawberry fruits. The expression data were determined by RT-qPCR and correspond to the mean of three biological replicates ± S.E normalized against controls. Differences between means were determined using two-way ANOVA and Tukey test. Asterisks indicate significant differences (* *p* < 0.05, ** *p* < 0.01, *** *p* < 0.001, and **** *p* < 0.0001). Triangles (▲) above bars represent significant differences between treatment and respective controls (*p* < 0.05).

The final steps of anthocyanin and PA biosynthesis in strawberry are controlled by the enzymes anthocyanidin synthase (ANS) and UDP glucose:flavonoid 3-*O*-glucosyl transferase (UFGT), and leucoanthocyanidin reductase (LAR) and anthocyanidin reductase (ANR), respectively. The expression patterns for the encoding genes for FaANS, FaUFGT, FaLAR, and FaANR were analyzed with the aim of verifying a possible coordination between the observed accumulation pattern of anthocyanin and PAs (Figure 3) and the expression profile of MBW-related genes (Figure 4). We noted that all treatments notably altered the expression profiles of anthocyanin and PA biosynthetic genes (Figure 6, Supplementary Table S9). The anthocyanin biosynthesis-related genes *FaANS* and *FaUFGT* exhibited similar expression patterns, considering all treatments at 24 and 48 h (Figure 6A,B). These genes showed a significant increment in their expression level in MeJA-treated fruit with respect to control and other treatments, the same as the increase in anthocyanin content detected in this treatment at 48 h (Figure 3A). Treatments with jarin-1 downregulated *FaANS* and *FaUFGT* expression at 48 h regarding MeJA treatment (Figure 6A,B).

With respect to the PA biosynthesis-related genes *FaLAR* and *FaANR*, their expression patterns showed differences mainly at 24 and 48 h under different treatments (Figure 6C,D). While *FaANR* expression level decreased in MeJA treatment with respect to its control and jarin-1 treatment at 24 h, *FaLAR* expression level increased compared to its control (Figure 6C,D). At 48 h, opposite patterns were observed. We noticed a direct relationship between the increased *FaANR* expression level and PA accumulation in MeJA + jarin-1 treatment at 48 h. This was not observed in the case of the *FaLAR* expression pattern at the same time (Figure 3B and Figure 6C,D).

In addition, the expression profile of *FaANR* in all treatments at 48 h was similar to those observed for *FaMYB9, FaMYB11*, *FabHLH33, FaJAR1.2*, and *FaJMT* in all treatments at 48 h and was related to PA accumulation in MeJA + jarin-1 treatment at 48 h (Figure 3B, Figure 4A–C, Figure 5 and Figure 6C).

During strawberry fruit development and ripening, we observed a downregulation pattern for *FaMYB9/11*, *FaANR*, and *FaLAR*, which fit with the PA accumulation pattern described during fruit developmental stages (Supplementary Figure S2). Conversely, an upregulation pattern was noticed for *FaMYB10*, *FaUFGT*, and *FaANS* and for the repressor encoding gene *FaMYB1* that matched the anthocyanin accumulation pattern during fruit development and ripening (Supplementary Figure S3A,B,D–F). In the case of *FabHLH3*, it exhibited constant expression levels from flowering to white-fruit stages and then decreased to the ripe stage (Supplementary Figure S3C). 

Finally, we analyzed key JA signaling-related genes such as *FaJAZ1* and *FaMYC2* to evaluate the effect of MeJA and jarin-1 treatments (Supplementary Figure S4). *FaMYC2* presented a downregulation pattern in MeJA treatment at 24 and 48 h and in MeJA + jarin-1 treatment at 48 h compared to controls (Supplementary Figure S4A). *FaJAZ1* exhibited upregulation in MeJA + jarin-1 treatment at 48 h (Supplementary Figure S4B).

In summary, we showed that the combination of exogenous MeJA application for up to 24 h with the addition of jarin-1 to strawberry fruit for up to 48 h activates PA biosynthesis, possibly through upregulation of *FaMYB9*, *FaMYB11*, *FabHLH33*, and *FaANR*. On the other hand, MeJA-induced anthocyanin accumulation is associated with upregulation of *FabHLH3*, *FaANS*, and *FaUFGT*.

## 3. Discussion

Proanthocyanidins (PAs) are among the most ubiquitous groups of plant phenolics distributed in plant-derived foods, such as fruits, and have several potential beneficial effects on human health [43]. Although several efforts have been made to reveal the steps of PA biosynthesis and its regulation in different species [15,44,45,46], there are still aspects to be uncovered. Otherwise, the phytohormone jasmonate (JA) has been associated with the accumulation of secondary metabolites through different JA-responsive transcription factors (TFs), including bHLH and MYB (reviewed by [47]). In apple, a mechanism for JA-induced biosynthesis of anthocyanin and PAs has been described, in which the JA signaling pathway could control the activity of the MBW complex [27].

In the present research, we report alterations on fruit firmness, weight, and skin color, PA and anthocyanin accumulation, and associated gene expression patterns when jarin-1, a JA-Ile biosynthesis inhibitor [41], was applied to MeJA-treated strawberry fruits. 

In the case of fruit weight, we report significant increases in both MeJA and jarin-1 treatments at different times (Supplementary Table S2). However, in terms of fruit weight changes, the effects of MeJA application on strawberry fruit do not show a clear trend. In this sense, Pérez et al. [37] reported a higher growth rate for strawberry (*F.* × *ananassa* cv. Camarosa) fruit treated with MeJA in an in vitro ripening system, which had weight gain with respect to control fruits. Inversely, Concha et al. [38] reported no significant differences in the weight of MeJA-treated *Fragaria chiloensis* fruit compared to controls, and a weight decrease in all treatments relative to the initial weight at 0 d was also shown [38]. In terms of fruit firmness, we observed a reduction in fruit firmness in both MeJA and jarin-1 treatments at 12 and 24 h, although a more pronounced decline was seen in jarin-1-treated fruits (Supplementary Table S2). Concha et al. [38] previously observed that the firmness of MeJA-treated *F. chiloensis* fruit was significantly higher from 48 to 120 h of treatment. In turn, Pérez et al. [37] reported that firmness of *F.* × *ananassa* (cv. Camarosa) fruits decreased following a sigmoidal curve, without differences between MeJA and control treatments. The differences in the reported weight and firmness gains or declines could reflect specific differences between species or cultivars and their behavior in in vitro ripening assays.

Color is a highly appreciated trait in strawberry fruit quality due to its relationship with flavonoid composition [48,49]. We confirmed previous results about the promoter role of exogenous MeJA in the acquisition of red coloration of strawberry fruit. Contrary to the uncertain effects of MeJA in fruit firmness and weight (Supplementary Table S2), to a certain extent, JA promotes color acquisition of strawberry fruit [33]. Exogenous MeJA applied in an in vitro ripening system increased fruit skin redness (higher a* values) in both *F. chiloensis* and *F.* × *ananassa* (cv. Aromas) [33,38], the same as is shown in the present research for *F.* × *ananassa* (cv. Albion) (Figure 2B). On the other hand, jarin-1 treatment showed an opposite effect to MeJA, since it decreased fruit redness (a* values) and increased fruit lightness (L* values) to a greater extent than MeJA (Figure 2A,B). We also confirmed previous results about MeJA-related anthocyanin accumulation observed in *Fragaria* species and cultivars [33,37,38], which involved a close relationship between red color acquisition and total anthocyanin accumulation patterns, as we observed in MeJA-treated fruits at 48 h (Figure 3A). MeJA application could have a stimulatory effect on anthocyanin biosynthesis in strawberry through the activation of JA signaling by an increase in JA-Ile biosynthesis [33]. In this sense, jarin-1 could inhibit FaJAR1 activity, as has been shown for Arabidopsis JAR1 [41], blocking JA signaling and thus anthocyanin accumulation, as was observed in the present research for the negative a* values and decreasing anthocyanin levels of jarin-1-treated fruits (Figure 2B and Figure 3A). In this regard, it was reported that application of jarin-1 inhibits anthocyanin accumulation induced by MeJA in both the wild-type Arabidopsis and *jar1-1* mutant [41]. Moreover, jarin-1 also decreases anthocyanin accumulation in *Cardamine hirsuta*, a species related to *A. thaliana* [41]. Until now, the present research is the first to report on the effect of jarin-1 in fleshy fruits, especially on anthocyanin and PA accumulation during fruit development.

A decreasing pattern of PA content during fruit development and ripening in different strawberry cultivars has been reported previously [13,14,16]. A lower equivalent catechin value was detected in MeJA treatment at 48 h, which suggests that this low PA content could be due to a redirection toward the anthocyanin biosynthesis pathway at the expense of PA biosynthesis (Figure 3). In turn, jarin-1 treatment caused a reverse effect, since it increased PA and decreased anthocyanin content (Figure 3). Similarly, the redirection between the anthocyanin and PA biosynthesis pathways has been reported in strawberry fruit by means of downregulation of genes encoding for the key biosynthetic enzymes FaUFGT and FaANR [18,50]. It is important to point out that for the strawberry ANS, UFGT, ANR and LAR enzymes 3, 37, 5, and 1 different coding sequences, respectively, exist in the *Fragaria vesca* genome (Supplementary Table S10), which is a subgenome of *F.* × *ananassa* [51]. This reveals that a variable number of genes encode for these enzymes in *F. vesca*. However, according to previous information on expression patterns reported for these genes in *F*. × *ananassa* fruit [13,52], just some of them show a relationship with the anthocyanin and PA accumulation pattern during fruit development (Supplementary Figures S2 and S3, Supplementary Table S10). In the present research, we used and analyzed the coding sequences corresponding to those gene family members that are closely related to the anthocyanin and PA biosynthesis (Supplementary Figures S2 and S3, Supplementary Table S10).

To investigate the possible activator role of jarin-1 on PA biosynthesis, we analyzed gene expression profiles of a set of genes encoding for key enzymes of anthocyanin and PA biosynthesis. In Arabidopsis, jarin-1 application inhibited the activity of the enzyme JAR1 and in turn decreased the expression of *LOX2* the encoding gene for lipoxygenase, a key enzyme for JA biosynthesis [41]. In the present research, we showed that application of jarin-1 to MeJA-treated fruits resulted in an increase of *FaJMT* and *FaJAR1.2* expression levels. Previously, Concha et al. [38] reported a significant increase in *FcJMT1* expression in *F. chiloensis* fruit treated with MeJA at 48 h, supporting a role for exogenous MeJA in the upregulation of the *JMT* gene. In regard to the increment of *FaJAR1.2* expression level, it is worth noting that jarin-1 is an enzymatic inhibitor, so an increase in *FaJAR1.2* gene expression does not necessarily mean an increase in JA-Ile levels, since post-translation regulation could take place, as has been suggested in JA-induced responses [53]. Although we did not register the JA-Ile level variations in jarin-1-treated samples, we observed a significant stimulation effect on PA accumulation by jarin-1 application that could act positively together with MeJA for the transcriptional regulation of key PA biosynthesis-related genes. Thus, we focused on establishing the effects of jarin-1 in combination with MeJA on the expression of the MBW complex– and PA biosynthesis-related genes.

Regulation of anthocyanin and PA biosynthesis is mainly controlled at the transcriptional level through the components of the MBW complex. In strawberry, FaMYB9/FaMYB11, FabHLH3, and FaTTG1 form a complex that upregulates the expression of genes encoding for FaANS, FaANR, and FaLAR, thereby increasing the PA content [15]. It seems that MYB TFs related to PA or anthocyanin biosynthesis can interact with bHLH3 or bHLH33 to form an MYB-bHLH complex [54]. We observed an increase in *FaMYB9, FaMYB11*, and *FabHLH33* expression levels in the MeJA + jarin-1 treatment, which coincided with higher PA content and *FaANR* expression levels in this treatment (Figure 3 and Figure 6). On the other hand, it was previously reported that for anthocyanin accumulation in apple [55] and peach [54], MYB10 uses bHLH3, rather than bHLH33, as a partner to activate the transcription of genes associated with anthocyanin biosynthesis. In our study, in observing the *FabHLH33* gene expression pattern, we suggest that this gene could be more related to PA than anthocyanin biosynthesis, in association with the FaMYB9 and FaMYB11 partners. Moreover, a coordinated downregulation pattern was observed in developing strawberry fruits (*F*. × *ananassa* cv. Aromas) between PA accumulation and *FaMYB9*, *FaMYB11*, *FaANR*, and *FaLAR* expression levels (Supplementary Figure S2). This observation allows the establishment of a relationship between gene expression profiles and related metabolite accumulations that we also observed in MeJA and jarin-1 treatments for changes in anthocyanin and PA contents and gene expression levels (Figure 3, Figure 4 and Figure 6). Overall, under the experimental conditions of the present research, we suggest that the TFs FaMYB9/FaMYB11 with FabHLH33, and likely not with FabHLH3, could upregulate PA biosynthesis through an increase in the *FaANR* rather than *FaLAR* transcriptional level. Moreover, the downregulation of *FaLAR* observed in the jarin-1 and MeJA + jarin-1 treatments at 48 h could imply a change in FaLAR activity and therefore a lower decrease in the proportion of trimeric PAs, as was observed for the minor reduction in mDP3 (%) in those treatments at 48 h compared to MeJA-treated fruit (Supplementary Table S5). Recently, it was described that LAR contributes to a certain extent to PA chain length in *Medicago* [46].

On the other hand, MeJA-treated fruit showed early upregulation of *FabHLH3* (Figure 4F). In the case of *FaMYB10*, we did not observe a clear induction under MeJA treatment, even though only MeJA-treated fruits showed a constant increase in anthocyanin accumulation (Figure 3A and Figure 4B). Previous reports established that MYB10 is clearly associated with anthocyanin biosynthesis. On transgenic strawberry plants with constitutive expression of *FaMYB10*, it was observed that an increase in *FaMYB10* expression caused higher anthocyanin biosynthesis levels [56]. Moreover, silencing of MYB10 in strawberry produced a clear decrease of anthocyanin content on the fruit receptacle [22]. In apple, Espley et al. [55] reported that transgenic plants that overexpressed *MYB10* had higher contents of anthocyanin and flavonoids. We suggest that JA could be regulating the anthocyanin biosynthesis in strawberry fruit via the FaMYB10-FabHLH3 complex. In apple calluses, An et al. [27] observed that the JA signaling pathway could control anthocyanin and PA biosynthesis through a repression of bHLH3 by JAZ proteins preventing the interaction with MYB partners. Under this model, when JA-Ile content rises, JAZ protein is degraded by the proteasome pathway, and then bHLH3 is released to form the MBW complex, recovering its transcriptional activity to activate downstream genes, finally leading to anthocyanin and PA biosynthesis [27].

In the case of the encoding gene for FaMYB1, which has been associated with a repressor role in anthocyanin biosynthesis in *F.* × *ananassa* [28,29], upregulation was observed in MeJA and MeJA + jarin-1 treatments at 48 h, even though a significantly high level was observed in MeJA + jarin-1 treatment (Figure 4E), suggesting that a synergistic action between MeJA and jarin-1 could be occurring to regulate *FaMYB1* expression. 

As the MYB TFs are a central component of the MBW regulatory complex, we isolated and analyzed the promoter regions of the MYB encoding genes. Phytohormone-related *cis* elements were found in each promoter region of the MYB encoding genes (Supplementary Figure S1, Supplementary Table S8). Remarkably, we found JA-responsive elements in all *FaMYB* promoter regions analyzed, such as the canonical G-box 5′-CACGTG-3′ [35,57,58] followed by one thymidine nucleotide (G-Box-T, [59]) in the *FaMYB1* promoter; the G-box variants 5′-AACGTG-3′ [35,57,58,60] and 5′-CACGTT-3′ [61] in the *FaMYB9* promoter and three different positions in the *FaMYB10* promoter; and 5′-CACATG-3′ [61] in the promoter of *FaMYB11*. These findings suggest that the JA signaling pathway could, at least in part, control the transcriptional activity of MWB complex-related *MYB* genes in strawberry.

Finally, with the aim of finding whether the JA signaling pathway is activated under the treatments of the present study, we analyzed the expression patterns of genes encoding for the TF MYC2 and repressor JAZ1, key components of JA signaling [62]. We observed downregulation of *FaMYC2* in MeJA treatments at 24 and 48 h, concomitant with the anthocyanin accumulation (Figure 3A, Supplementary Figure S4A). In this regard, contrasting results were previously shown in Arabidopsis. First, Shan et al. [34] observed that the *MYC2* mutant (*atmyc2-2*) and wild-type plants showed anthocyanin accumulation induced by JA, which suggests that MYC2 would not have a significant effect on anthocyanin accumulation induced by JA. Nevertheless, Niu et al. [36] reported that overexpressing either *MYC2*, *MYC3* or *MYC4* Arabidopsis seedlings accumulated higher anthocyanin content than wild-type plants. In strawberry fruit, more evidence is needed to assign a role for FaMYC2 in JA-induced anthocyanin accumulation. In the case of *FaJAZ1*, upregulation was observed in MeJA + jarin-1 treatment at 48 h that could be related to the PA accumulation with this treatment at this time (Figure 3B, Supplementary Figure S4B). We recently showed that *FaMYC2* and *FaJAZ1* were upregulated up to 6 h and 30 min, respectively, under MeJA treatment [16], suggesting that at least *FaMYC2* is upregulated at early times and downregulated at 24 and 48 h of MeJA treatment.

## 4. Materials and Methods 

### 4.1. Plant Material, Experimental Design, and Treatments

*Fragaria* × *ananassa* (cv. Albion) fruits at the large green stage, of similar size and without external damage, were harvested according to Garrido-Bigotes et al. [33] from plants grown in a commercial field at Pelluhue, Maule Region, Chile (latitude 35°47′49′′ S; longitude 72°33′22” W). After harvest, fruit peduncles were cut at 3.5 cm and then peduncles with intact fruits were immersed in an incubation solution (88 mM sucrose and 1 mM hydroxyquinoline hemisulfate (HQS)). The samples were immediately transported to the laboratory at Universidad de Talca (Talca, Chile, lat. 35°24′20′′ S; long. 71°38′9′′ W), and the fruits were transferred to an in vitro ripening system according to Perkins-Veazie and Huber [42] and Concha et al. [38]. Fruits in the incubation solution that were not transferred to the in vitro ripening system were considered as untreated (0 h) fruit (Scheme 1).

Briefly, the in vitro fruit ripening system consisted of the growth and development of picked fruits in a nutritious solution under controlled environmental conditions (Scheme 1). In a growth chamber, the fruits were transferred to sterile microfuge tubes (1.5 mL) with the peduncles immersed in the different treatment solutions for 12, 24, and 48 h, maintained at 24 °C under a 16 h photoperiod. Treatment solutions were 100 µM methyl jasmonate (Sigma Aldrich, St. Louis, MO, USA) in incubation solution (MeJA treatment); 60 µM jarin-1 (Analyticon Discovery GmbH, Potsdam, Germany) in incubation solution plus 19.9 µM dimethyl sulfoxide (DMSO) (jarin-1 treatment); and MeJA treatment up to 24 h and then transferred to jarin-1 solution up to 48 h (MeJA + jarin-1 treatment). The corresponding control solutions consisted of incubation solution (Control 1) and incubation solution plus DMSO (Control 2). MeJA concentration was selected according to Concha et al. [38]. In the case of jarin-1, we previously tested 30 and 60 μM concentrations in strawberry fruit and found that the latter resulted in significant differences in fruit firmness and color compared to control treated fruits (data not shown). 

The experiment was arranged in a randomized design to account for spatial variability. Each individual fruit was considered as a biological replicate. Six fruits were collected at 12, 24, and 48 h for each treatment and control and assessed for fruit quality. Then, 3 fruits were analyzed for anthocyanin and proanthocyanidin contents and 3 for gene expression. Values for MeJA-, jarin-1–, and MeJA + jarin-1-treated fruits under the parameters for each treatment time were calculated with respect to Control 1, Control 2, and the average of Control 1 and Control 2 values, respectively.

### 4.2. Fruit Quality Assessments 

Fruits from each treatment were weighed, and their firmness and color were estimated. Firmness of treated fruits was measured using a texture analyzer (CT3 model, Brookfield Engineering, Middleboro, MA, USA) with a TA-39 probe, a 3 mm target value, and a 1 mm/s test speed. Color changes on treated fruit skin were measured using a colorimeter (model CR-400, Konica Minolta, Tokyo, Japan) and expressed according to the CIELAB scale (L*, a*, b*) along with the dimensions of color chroma and hue angle (h°). L*, a*, and b* indicate lightness, chromaticity on a green (−) to red (+) axis, and on a blue (−) to yellow (+) axis, respectively. Chroma (chroma = (a*^2^ + b*^2^)^1/2^) and hue angle (h° = arctan (b*/a*)) were calculated from numerical values of a* and b* as previously reported [63]. For firmness and color measurement, 2 technical replicates were made for each treated fruit on the equatorial side and were measured at 12, 24, and 48 h of treatment. For all fruit quality assessments, the results are expressed as difference (delta, Δ) between the values of treatments and their respective controls at each time point.

### 4.3. Anthocyanin Quantification

Total anthocyanin content (TAC) was quantified by the pH differential method performed according to Lee et al. [64] and Debnath and Ricard [65]. Fruit skin without achenes (150 mg) was ground with liquid nitrogen, homogenized with 1.5 mL of absolute ethanol and 1.5 N HCl (85:15 *v*/*v*), incubated overnight at 4 °C, and centrifuged for 10 min at 12,000 rpm at 4 °C. After that, 2 aliquots from the aqueous phase of each sample were diluted (1:4) with 2 different buffers: a pH 1 buffer (0.025 M KCl) and a pH 4.5 buffer (0.4 M sodium acetate). Finally, the sample absorbances were quantified at 516 and 700 nm. Buffers for each pH were used as blanks. The anthocyanin content of each sample was calculated based on the Lambert–Beer law, using the coefficient of molar extinction for the pelargonidin-3-glucoside (31,620 M^−1^·cm^−1^) reported by Swain [66]. Results are expressed as difference (delta, Δ) between the TAC (micrograms pelargonidin-3-glucoside equivalent per gram of fresh weight) values of treatments and their respective controls at each time point.

### 4.4. Proanthocyanidin Quantification and Total PA Polymerization Analysis 

For each treatment at each sampling time point, a bulk of 3 g of strawberry fruits without achenes was ground with liquid nitrogen, homogenized in 9 mL of 80% acetone, and sonicated at room temperature for 30 min. Total proanthocyanidin content (TPC) was quantified according to Prior et al. [67]. The reaction mixture contained 70 μL of diluted (1:50) samples and 210 μL 0.1% 4-dimethylaminocinnamaldehyde (DMACA) in 80% acidified ethanol. Ethanol (80% acidified) was used as a blank. The plate was incubated for 20 min at room temperature and measured at 640 nm using a Biotek ELISA plate reader equipped with the Gen5 software package (Biotek Instruments Inc., Winooski, VT, USA). TPC was calculated by linear regression based on the absorbance values of a standard curve of catechin (0–15.625 μg mL^−1^). Results are expressed as difference (delta, Δ) between TPC (micrograms catechin equivalent per gram of fresh weight) values of treatments and their respective controls at each time point. Mean degree of polymerization (mDP) of total PA was characterized by hydrophilic interaction chromatography (HILIC-HPLC) according to Pastene et al. [68]. 

### 4.5. Molecular Analysis

#### 4.5.1. RNA Isolation

Total RNA isolation from treated fruits of *F.* × *ananassa* (cv. Albion) was achieved by combining the RNeasy Plus Mini Kit (Qiagen, Hilden, Germany) and the cetyltrimethylammonium bromide (CTAB) method described by Liao et al. [69] with some modifications. Three biological replicates were used for each treatment. For cDNA, 1 µg of total RNA was treated with DNase I (Fermentas, Waltham, MA, USA) and the synthesis was performed using the RevertAid H Minus First Strand cDNA Synthesis Kit (Thermo Scientific, Waltham, MA, USA) according to the manufacturer’s instructions. 

#### 4.5.2. Genes Analyzed

We analyzed the expression level of genes previously reported as related to the following processes or pathways: JAs biosynthesis (*FaJMT* and *FaJAR1.2* [33,70]), JA signaling (*FaMYC2* and *FaJAZ1* [16]), MBW complex (*FaMYB9*, *FaMYB10*, *FaMYB11*, *FabHLH3*, and *FabHLH33* and repressor *FaMYB1* [15]), and anthocyanin and PA biosynthetic genes (*FaANS*, *FaUFGT*, *FaANR*, and *FaLAR* [15,71,72], Supplementary Table S10). Glyceraldehyde 3-phosphate dehydrogenase (*FaGAPDH* [70]) encoding gene was used as the reference housekeeping gene. Primer sequences are described in Supplementary Table S11. Primers for *FaMYB10* and *FabHLH33* were designed in the present research and the other primer sequences were as previously reported [15,16,33,70,71]. 

#### 4.5.3. Reverse Transcription-qPCR (RT-qPCR) Analysis

RT-qPCR analysis was performed using the KAPA SYBR^®^FAST qPCR Kit (Kapa Biosystems, Wilmington, MA, USA), according to the manufacturer’s instructions, and the PikoReal Real-Time PCR System (Thermo Scientific, USA). The PCR conditions were: 94 °C for 10 min; 40 cycles at 94 °C for 15 s, 60 °C for 15 s, and 72 °C for 15 s. Each reaction was repeated 3 times, using water as negative control. The relative gene expression levels correspond to the mean of 3 biological replicates normalized against the expression level of the housekeeping gene using untreated fruits (0 h) (Scheme 1) as the calibrator sample and assigned a nominal value of 1, according to the 2^−ΔΔ*C*t^ method [73]. Relative gene expression levels were expressed as fold changes (Δ fold), between the fold change of each treatment and their respective controls at each time point:

Relative gene expression (Δ fold) = Fold change of Treatment − Fold change of Control

#### 4.5.4. Promoter Isolation and In Silico Analysis

Promoter regions of *F.* × *ananassa MYB* genes (*FaMYB9* (GenBank JQ989281), *FaMYB10* (GenBank MG456859), *FaMYB11* (GenBank JQ989282), and the repressor *FaMYB1* (GenBank AF401220)) were isolated by a PCR-based method using the *Fragaria vesca* genome as reference. mRNA sequences of *MYB* genes were used to search orthologous genes in the *F. vesca* genome (Genome, National Center for Biotechnology Information (NCBI), https://www.ncbi.nlm.nih.gov/genome/3314) by means of the BLAST genome search tool (NCBI, https://blast.ncbi.nlm.nih.gov/Blast.cgi) (Supplementary Table S12). Primers were designed using Primer3, and the target region for PCR amplification was set from −2000 to +100 bp for each gene (Supplementary Table S13).

Genomic DNA extraction from leaves of *F.* × *ananassa* (cv. Aromas) was performed by the CTAB method [74] with some modifications. PCR was carried out using Phusion DNA polymerase (Thermo Scientific, USA), and the conditions are described in Supplementary Table S14. Fragment length of each putative promoter was confirmed by 1% (*w*/*v*) agarose gel and sequenced on both strands by primer walking (Macrogen, Seoul, Korea). Sequences were assembled using SeqMan Pro software (version 7.1.0, DNASTAR, Madison, WI, USA) and their identity was confirmed by BLAST Genome.

Identification of putative cis elements in the sequenced promoters was performed using the PlantCARE database (http://bioinformatics.psb.ugent.be/webtools/plantcare/html/) [75]. We also analyzed jasmonate (JA)-responsive elements in each promoter according to G-box and its variants, described and characterized previously with experimental approaches [35,57,58,59,60,61].

### 4.6. Statistical Analysis

Data were analyzed by one-way analysis of variance (ANOVA) using Infostat software (version 2015). LDS test and Tukey post hoc test were used to evaluate significance for physiological data (firmness, weight, color, TAC, TPC, and mDP) and RT-qPCR gene expression analysis, respectively. Values of *p* ≤ 0.05 were considered statistically significant.

## 5. Conclusions

The regulation of PA and anthocyanin biosynthesis in fleshy fruits is a complex process due to the participation of several molecular components. In the present research, we found a connection between the JA pathway and PA and anthocyanin biosynthesis in strawberry fruit, although further investigation is needed to decipher how the MeJA and jarin-1 interaction works at the molecular level and what the specific roles of the JA signaling pathway-associated components are in anthocyanin and PA biosynthesis. This work provides evidence for potential uses of hormone inhibitors for the study of several biochemical pathways during fruit ripening and for chemical manipulation of the flavonoid pathway, especially in strawberry, which could enrich the nutritional quality of this fruit. 

## Supplementary Materials

The following are available online at http://www.mdpi.com/1420-3049/23/6/1433/s1: Figure S1: Putative phytohormone-responsive elements distribution in the promoter regions of *FaMYB* genes. Roman numerals indicate phytohormones: I, JA; II, ABA; III, ET; IV, GA; and V, SA. Arabic numerals indicate the distance from the 5′ UTR in bp. Motif locations and features is according to Table S8. JA, jasmonate; ABA, abscisic acid; ET, ethylene; GA, gibberellin; SA, salicylic acid. Figure S2: (A) Changes in proanthocyanidin (PA) content and expression levels of the PAs biosynthesis-related genes (B) *FaMYB9*, (C) *FaMYB11*, (D) *FaANR*, and (E) *FaLAR* during fruit development and ripening of strawberry (*Fragaria* × *ananassa* cv. Aromas) fruits. Developmental stages correspond to flowering (F), small green (SG), large green (LG), white (W), turning (T), 50% red receptacle (50%R), and 100% red receptacle (100%R) according to Garrido-Bigotes et al. 2018 [33]. Methodology for PA quantification and gene expression analysis is described in the Materials and Methods section. Fruits from the white developmental stage were used as the calibrator sample. Relative gene expression levels were expressed as a difference between relative gene expression levels in each developmental stage and white stage. PA content evolution in panel A was previously reported by Garrido-Bigotes et al. 2018 [16]. Figure S3: (A) Changes in anthocyanin content and expression levels of the anthocyanin biosynthesis-related genes (B) *FaMYB1*, (C) *FabHLH3*, (D) *FaMYB10*, (E) *FaANS*, and (F) *FaUFGT* during fruit development and ripening of strawberry (*Fragaria* × *ananassa* cv. Aromas) fruits. Developmental stages correspond to flowering (F), small green (SG), large green (LG), white (W), turning (T), 50% red receptacle (50%R), and 100% red receptacle (100%R) according to Garrido-Bigotes et al. 2018 [33]. Methodology for anthocyanin quantification and gene expression analysis is described in the Materials and Methods section. Fruits from the white developmental stage were used as the calibrator sample. Relative gene expression levels were expressed as a difference between relative gene expression levels in each developmental stage and white stage. Anthocyanin content evolution in panel A was previously reported by Garrido-Bigotes et al. 2018 [16]. Figure S4: Changes in relative expression levels of (A) *FaMYC2* and (B) *FaJAZ1* genes at different time points under treatment in comparison to respective controls during the in vitro ripening of strawberry fruits. The expression data were determined by RT-qPCR and correspond to the mean of three biological replicates ± S.E normalized against controls. Differences between means were determined using two-way ANOVA and Tukey test. Asterisks indicate significant difference (* *p* < 0.05 and **** *p* <0.0001). Triangles (▲) above bars represent significant differences between treatments and their respective controls (*p* < 0.05). Table S1: Changes (Δ) in fruit skin color according to CIEL*a*b* scale, chroma, and hue at different time points under treatments during the in vitro ripening of strawberry fruits. Table S2: Changes (Δ) in fruit firmness (N) and weight (g) at different treatments during the in vitro ripening of strawberry fruits. Table S3: Changes (Δ) in total anthocyanin content (TAC) at different treatments during the in vitro ripening of strawberry fruits. Table S4: Changes (Δ) in total proanthocyanidin content (TPC) at different treatments during the in vitro ripening of strawberry fruits. Table S5: Changes (Δ) in total proanthocyanidin mean degree of polymerization (mDP, %) at different treatments during the in vitro ripening of strawberry fruits. Table S6: Changes in relative expression levels of MBW complex-related genes at different treatments during the in vitro ripening of strawberry fruits. Table S7: Changes (Δ) in relative expression levels of jasmonate pathway-related genes at different treatments during the in vitro ripening of strawberry fruits. Table S8: Putative phytohormone-responsive elements found within the analyzed region of *FaMYB9*, *FaMYB10*, *FaMYB11*, and *FaMYB1* promoters. Table S9: Changes (Δ) in relative expression levels of anthocyanin and proanthocyanidin biosynthesis-related genes at different time points under treatments during the in vitro ripening of strawberry fruits. Table S10: Genomic data and expression and expression patterns during fruit development and ripening for anthocyanidin synthase (*ANS*), UDP glucose:flavonoid 3-*O*-glucosyltransferase (*UFGT*), anthocyanidin reductase (*ANR*), and leucoanthocyanidin reductase (*LAR*) gene family in *Fragaria vesca* and *Fragaria* × *ananassa*, respectively. Table S11: Primer sequences used for RT-qPCR analysis. Table S12: Predicted sequences of *Fragaria* × *ananassa MYB* genes on *Fragaria vesca* genome. Table S13: Primer sequences used for promoter isolation of *MYB* genes and primer walking sequencing. Table S14: PCR conditions for amplification of *MYB* promoters.
