# Supplementary material for: Application of a JA-Ile Biosynthesis Inhibitor to Methyl Jasmonate-Treated Strawberry Fruit Induces Upregulation of Specific MBW Complex-Related Genes and Accumulation of Proanthocyanidins
Source: Molecules. 2018 Jun 13;23(6):1433. doi: 10.3390/molecules23061433 (PMC6100305; doi:10.3390/molecules23061433)
Supplement: Supplementary file 1 [file molecules-23-01433-s001.zip › Table S5.docx]

**Table S5**. Changes (Δ) in total proanthocyanidin mean degree of polymerization (mDP, %) at different treatments during the *in vitro* ripening of strawberry fruits.

| **Time** | **Treatment ^1^** | **Mean degree of polymerization (mDP, %)** | | | |
| --- | --- | --- | --- | --- | --- |
|  |  | **Δ mDP1 (%)** | **Δ mDP2 (%)** | **Δ mDP3 (%)** | **Δ mDP≥4 (%)** |
| 0 h | Untreated | 20.53 ± 0.39 | 9.27 ± 0.03 | 9.03 ± 0.1 | 57.93 ± 0.32 |
| 12 h | MeJA | 1.77 ± 0.84a ^2^ | 0.33 ± 0.06a | -0.67 ± 0.1a | -1.07 ± 0.8a |
|  |  | (21.5 - 19.73) | (9.3 - 8.97) | (8.03 - 8.7) | (57.43 - 58.5) |
|  | jarin-1 | -1.39 ± 1.56a | -0.07 ± 0.42a | -0.65 ± 0.45a | 3.23 ± 2.3a |
|  |  | (18.61 - 20) | (9.4 - 9.47) | (8.31 - 8.96) | (60.93 - 57.7) |
| 24 h | MeJA | -1.10 ± 0.50a | -0.23 ± 0.23a | -0.15 ± 0.17a | 2.27 ± 0.06a |
|  |  | (18.07 - 19.17) | (8.93 - 9.17) | (8.72 - 8.87) | (61.13 - 58.86) |
|  | jarin-1 | -3.13 ± 1.35a | -0.70 ± 0.55a | -0.83 ± 0.58a | 5.57 ± 2.34a |
|  |  | (19.9 - 23.03) | (10.05 - 10.75) | (9.03 - 9.87) | (57.77 - 52.2) |
| 48 h | MeJA | -3.60 ± 0.64a | -0.67 ± 0.22a | -1.77 ± 0.62a | 5.77 ± 1.66a |
|  |  | (20.03 - 23.63) | (10.17 - 10.83) | (9.7 - 11.47) | (56.53 - 50.77) |
|  | jarin-1 | -3.03 ± 0.32a | -0.67 ± 0.13a | -0.27 ± 0.15b | 4.9 ± 0.18a |
|  |  | (18.6 - 21.63) | (10.27 - 10.93) | (9.63 - 9.9) | (58.03 - 53.13) |
|  | MeJA+jarin-1 ^3^ | -2.96 ± 0.71a | -0.38 ± 0.10a | -0.19 ± 0.31b | 3.72 ± 0.30a |
|  |  | (19.67 -22.63) | (10.49 - 10.87) | (10.51 - 10.69) | (55.67 - 51.95) |

^1^ MeJA and jarin-1 treatments involved the application of 100 μM MeJA and 60 μM jarin-1, and measurements were performed at 12, 24, and 48 h. MeJA+jarin-1 treatment involved the addition of 60 μM jarin-1 to 100 μM MeJA solution at 24 h and the measurements were performed at 48 h. For details, see Scheme 1.

^2^ Values (delta, Δ) are mean of three biological replicates ± S.E normalized. Delta was calculated as the difference between the mean of treatments and their respective controls at each time (Treatment – Control). Lowercase letters correspond to significant differences between treatments at the same time. Asterisks indicate significant differences with each control treatment. Differences were considered statistically significant at p≥0.05 (LSD test).
