# Supplementary material for: Application of a JA-Ile Biosynthesis Inhibitor to Methyl Jasmonate-Treated Strawberry Fruit Induces Upregulation of Specific MBW Complex-Related Genes and Accumulation of Proanthocyanidins
Source: Molecules. 2018 Jun 13;23(6):1433. doi: 10.3390/molecules23061433 (PMC6100305; doi:10.3390/molecules23061433)
Supplement: Supplementary file 1 [file molecules-23-01433-s001.zip › Table S9.docx]

**Table S9.** Changes (Δ) in relative expression levels of anthocyanin and proanthocyanidin biosynthesis-related genes at different treatments during the *in vitro* ripening of strawberry fruits.

| **Time** | **Treatment ^1^** | Δ **Relative expression** | | | |
| --- | --- | --- | --- | --- | --- |
|  |  | ***FaANS*** | ***FaUFGT*** | ***FaANR*** | ***FaLAR*** |
| 12 h | MeJA | -2.26 ± 0.95a ^2^ | 3.33 ± 5.81a | 0.22 ± 0.32a | 0.55 ± 0.60a |
|  |  | (2.65 – 5.53) | (4.59 - 1.26) | (0.90 - 0.69) | (1.62 - 1.07) |
|  | jarin-1 | -1.99 ± 0.00a | 0.67 ± 1.79a | -0.16 ± 0.04a | 1.33 ± 0.61a |
|  |  | (3.53 – 5.52) | (2.63 - 1.95) | (0.72 - 0.88) | (2.00 - 0.67) |
| 24 h | MeJA | 21.79 ± 0.44b* | 701.79 ± 63.92b* | -0.63 ± 0.10a | 3.97 ± 1.57a* |
|  |  | (28.43 – 6.64) | (711.24 - 9.45) | (0.32 - 0.95) | (6.72 - 2.75) |
|  | jarin-1 | 11.96 ± 5.29a* | 242.09 ± 46.49a* | -0.13 ± 0.16b* | 2.36 ± 1.07a |
|  |  | (15.66 – 3.71) | (268.32 - 26.23) | (0.45 - 0.58) | (5.06 - 3.08) |
| 48 h | MeJA | 18.20 ± 5.18b* | 787.78 ± 158.39b* | 0.00 ± 0.19a | 1.61 ± 0.11b* |
|  |  | (27.32 – 9.12) | (911.97 - 173.10) | (0.67 - 0.59) | (5.10 - 3.49) |
|  | jarin-1 | -7.67 ± 6.31a | -223.46 ± 76.40a | 0.16 ± 0.18a | -0.95 ± 1.00a |
|  |  | (15.05 – 22.72) | (421.67 - 645.13) | (0.74 - 0.58) | (4.96 - 5.91) |
|  | MeJA+jarin-1 | -9.22 ± 1.97a | -230.71 ± 100.86a | 1.41 ± 0.08b* | -2.13 ± 0.70a* |
|  |  | (8.06 – 17.28) | (127.22 - 361.91) | (2.00 - 0.59) | (2.82 - 4.94) |

^1^ MeJA and jarin-1 treatments involved the application of 100 μM MeJA and 60 μM jarin-1, and measurements were performed at 12, 24, and 48 h. MeJA+jarin-1 treatment involved the addition of 60 μM jarin-1 to 100 μM MeJA solution at 24 h and measurements were performed at 48 h. For details, see Scheme 1.

^2^ Values (delta, Δ) are mean of three biological replicates ± S.E normalized. Delta was calculated as the difference between the mean of treatments and their respective controls at each time (Treatment – Control). Lowercase letters correspond to significant differences between treatments at the same time. Asterisks indicate significant differences with each control treatment. Differences were considered statistically significant at p≥0.05 (LSD test).
