# Supplementary material for: Application of a JA-Ile Biosynthesis Inhibitor to Methyl Jasmonate-Treated Strawberry Fruit Induces Upregulation of Specific MBW Complex-Related Genes and Accumulation of Proanthocyanidins
Source: Molecules. 2018 Jun 13;23(6):1433. doi: 10.3390/molecules23061433 (PMC6100305; doi:10.3390/molecules23061433)
Supplement: Supplementary file 1 [file molecules-23-01433-s001.zip › Table S8.docx]

**Table S8.** Putative phytohormone-responsive elements found within the analyzed region of *FaMYB9*, *FaMYB10*, *FaMYB11*, and *FaMYB1* promoters.

| **Hormone ^2^** | **Motif ^3^** | ***pFaMYB1* (1500) ^1^** | | | ***pFaMYB9* (1900)** | | | ***pFaMYB10* (1700)** | | | ***pFaMYB11* (1700)** | | |
| --- | --- | --- | --- | --- | --- | --- | --- | --- | --- | --- | --- | --- | --- |
|  |  | **Sequence** | **Strand** | **Distance from 5’UTR ^4^** | **Sequence** | **Strand** | **Distance**  **from 5’UTR** | **Sequence** | **Strand** | **Distance**  **from 5’UTR** | **Sequence** | **Strand** | **Distance from 5’UTR** |
| JA | G-box ^5^ | CACGTGT | + | -1316 | AACGTG | - | -1806 | CACGTT | + | -1606 | CACATG | - | -1076 |
|  |  |  |  |  | CACGTT | + | -686 | CACGTT | + | -299 |  |  |  |
|  |  |  |  |  |  |  |  | CACGTT | + | -1548 |  |  |  |
|  | CGTCA-motif | CGTCA | - | -1132 |  |  |  | CGTCA | + | -806 | CGTCA | + | -515 |
|  |  | CGTCA | + | -427 |  |  |  | CGTCA | + | -770 |  |  |  |
|  |  | CGTCA | + | -1129 |  |  |  |  |  |  |  |  |  |
|  | TGACG-motif | TGACG | + | -1132 |  |  |  | TGACG | - | -806 | TGACG | - | -515 |
|  |  | TGACG | - | -427 |  |  |  | TGACG | - | -770 |  |  |  |
|  |  | TGACG | - | -1129 |  |  |  |  |  |  |  |  |  |
| ABA | ABRE | TACGTG | + | -285 | TACGTG | + | -1892 | TACGGTC | - | -346 | TACGTG | - | -652 |
|  |  | TACGTG | - | -279 | CGTACGTGCA | - | -1332 |  |  |  |  |  |  |
|  |  | CGTACGTGCA | - | -281 |  |  |  |  |  |  |  |  |  |
|  |  | CGTACGTGCA | + | -287 |  |  |  |  |  |  |  |  |  |
|  | IIb | CCGCCGCGCT | + | -1493 |  |  |  |  |  |  |  |  |  |
| ET | ERE |  |  |  | ATTTCAAA | + | -1644 |  |  |  | ATTTCAAA | + | -1319 |
| GA | GARE | AAACAGA | + | -939 |  |  |  |  |  |  |  |  |  |
|  |  | AAACAGA | + | -866 |  |  |  |  |  |  |  |  |  |
|  | P-box | CCTTTTG | - | -1323 |  |  |  |  |  |  |  |  |  |
|  |  | CCTTTTG | - | -1248 |  |  |  |  |  |  |  |  |  |
| SA | TCA-element | CCATCTTTTT | + | -1201 | CCATCTTTTT | + | -1543 | CCATCTTTTT | - | -1253 | GAGAAGAATA | - | -1250 |
|  |  | GAGAAGAATA | - | -229 | GAGAAGAATA | + | -1709 |  |  |  |  |  |  |

Identification of putative *cis* elements in promoter regions was performed using PlantCARE database (http://bioinformatics.psb.ugent.be/webtools/plantcare/html/;[75], and suitable references for G-box.

^1^ The sizes of the promoter regions of each gene (bp) are indicated in brackets.

^2^ Phytohormones included jasmonate (JA), abscisic acid (ABA), ethylene (ET), gibberellin (GA), and salicylic acid (SA).

^3^ Motifs included abscisic acid responsive element (ABRE), ethylene-responsive element (ERE), and gibberellin-responsive element (GARE).

^4^ 5’UTR: 5’ untranslated region.

^5^ G-box and its variants as follows: CACGTGT [59], AACGTG [35,57,58,60], CACGTT [61], and CACATG [61].
