# Supplementary material for: Application of a JA-Ile Biosynthesis Inhibitor to Methyl Jasmonate-Treated Strawberry Fruit Induces Upregulation of Specific MBW Complex-Related Genes and Accumulation of Proanthocyanidins
Source: Molecules. 2018 Jun 13;23(6):1433. doi: 10.3390/molecules23061433 (PMC6100305; doi:10.3390/molecules23061433)
Supplement: Supplementary file 1 [file molecules-23-01433-s001.zip › Table S4.docx]

**Table S4.** Changes (Δ) in total proanthocyanidin content (TPC) at different treatments during the *in vitro* ripening of strawberry fruits.

| **Treatment ^2^** | **Δ TPC ^1^ (µg g^−1^FW)** | | |
| --- | --- | --- | --- |
|  | **0 h** | | |
| **Untreated** | 494.04 ± 0.58 | | |
| **Treated** | **12 h** | **24 h** | **48 h** |
| MeJA | -93.08 ± 7.97a* ^3^  (465.44 - 558.51) | 37.64 ± 6.73b*  (537.43 - 499.79) | -151.38 ± 13.20a*  (457.64 - 609.02) |
| jarin-1 | -58.04 ± 0.40b*  (425.33 - 483.37) | -28.74 ± 13.91a  (474.47 - 503.21) | 44.80 ± 7.35b*  (519.00 - 474.20) |
| MeJA+jarin-1 | --- | --- | 63.37 ± 8.77b*  (584.98 - 521.60) |

^1^ TPC was quantified as µg of catechin equivalent per gram of fresh weight (FW).

^2^ MeJA and jarin-1 treatments involved the application of 100 μM MeJA and 60 μM jarin-1, and measurements were performed at 12, 24, and 48 h. MeJA+jarin-1 treatment involved the addition of 60 μM jarin-1 to 100 μM MeJA solution at 24 h and measurements were performed at 48 h. For details, see Scheme 1.

^3^ Values (delta, Δ) are mean of three biological replicates ± S.E normalized. Delta was calculated as the difference between the mean of treatments and their respective controls at each time (Treatment – Control). Lowercase letters correspond to significant differences between treatments at the same time. Asterisks indicate significant differences with each control treatment. Differences were considered statistically significant at p≥0.05 (LSD test).
