# Supplementary material for: Application of a JA-Ile Biosynthesis Inhibitor to Methyl Jasmonate-Treated Strawberry Fruit Induces Upregulation of Specific MBW Complex-Related Genes and Accumulation of Proanthocyanidins
Source: Molecules. 2018 Jun 13;23(6):1433. doi: 10.3390/molecules23061433 (PMC6100305; doi:10.3390/molecules23061433)
Supplement: Supplementary file 1 [file molecules-23-01433-s001.zip › Table S3.docx]

**Table S3.** Changes (Δ) in total anthocyanin content (TAC) at different treatments during the *in vitro* ripening of strawberry fruits.

| **Treatment ^2^** | **Δ TAC ^1^ (μg g^−1^FW)** | | |
| --- | --- | --- | --- |
|  | **0 h** | | |
| **Untreated** | 4.46 ± 1.36 | | |
| **Treated** | **12 h** | **24 h** | **48 h** |
| MeJA | -1.3 ± 1.4a ^3^  (6.1 – 7.3) | 5.6 ± 0.8a  (12.2 - 6.6) | 33.7 ± 23.3b  (52.5 – 18.8) |
| jarin-1 | -2.8 ± 0.3a  (1.8 – 4.6) | -3.5 ± 3.4a  (3.7 – 7.2) | -0.035 ± 0.8a  (4.2 – 4.2) |
| MeJA+jarin-1 | --- | --- | -3.4 ± 2.8a  (8.1 – 11.5) |

^1^ TAC was quantified as total μg of pelargonidin 3-glucoside equivalent per gram of fresh weight (FW).

^2^ MeJA and jarin-1 treatments involved the application of 100 μM MeJA and 60 μM jarin-1, and measurements were performed at 12, 24, and 48 h. MeJA+jarin-1 treatment involved the addition of 60 μM jarin-1 to 100 μM MeJA solution at 24 h and measurements were performed at 48 h. For details, see Scheme 1.

^3^ Values (delta, Δ) are mean of three biological replicates ± S.E normalized. Delta was calculated as the difference between the mean of treatments and their respective controls at each time (Treatment – Control). Lowercase letters correspond to significant differences between treatments at the same time. Asterisks indicate significant differences with each control treatment. Differences were considered statistically significant at p≥0.05 (LSD test).
