# Supplementary material for: Application of a JA-Ile Biosynthesis Inhibitor to Methyl Jasmonate-Treated Strawberry Fruit Induces Upregulation of Specific MBW Complex-Related Genes and Accumulation of Proanthocyanidins
Source: Molecules. 2018 Jun 13;23(6):1433. doi: 10.3390/molecules23061433 (PMC6100305; doi:10.3390/molecules23061433)
Supplement: Supplementary file 1 [file molecules-23-01433-s001.zip › Table S11.docx]

**Table S11**. Primer sequences used for RT-qPCR analysis.

| **Gene ^1^** | **Forward (5´→3´)** | **Reverse (5´→3´)** | **Reference** | **GenBank** ^2^ |
| --- | --- | --- | --- | --- |
| *FaJMT* | AATAAGCAGCGGCGAGCGAGTAGC | AAGCGATCACTGACGAGCTCTGCG | Preuß et al. 2014 [70] | XM_004291805 |
| *FaJAR1.2* | GGTGTGCCATTGGTTAGTGC | CACCCCAATCCAGCTCTCAG | Garrido-Bigotes et al. 2018 [33] | XM_004304072 |
| *FaMYC2* | AGGGGATCCTGTCGTTTACC | TTCGGGGTCCACAACTCTAC | Garrido-Bigotes et al. 2018 [16] | XM_004300191 |
| *FaJAZ1* | TGGGAGATCTGAACCTCGTC | TTCCTCGGTTTCTCCATCAC | Garrido-Bigotes et al. 2018 [16] | MF511104 |
| *FaMYB9* | GGTCAACATCCACTACCGCATTAG | CCAACTGGGTTTGAGGAACTTG | Schaart et al. 2013 [15] | JQ989281 |
| *FaMYB10* | GTGTGAGAAAAGGTGCATGGAC | TGCCATTTGCCTTCTCCATG | This research | MG456859 |
| *FaMYB11* | CAATGACCATCATGAGCTGCA | TCGATGATAGGAGCCATGGAT | Schaart et al. 2013 [15] | JQ989282 |
| *FabHLH3* | TGTGCCCTTCTTGCACACAA | GACGACTTCGGCGAATTCTC | Schaart et al. 2013 [15] | JQ989284 |
| *FabHLH33* | AGGCAAGAGCAGCAGAAATG | TCCGCTTGTTTATCGAAGGC | This research | JQ989286 |
| *FaMYB1* | GCAACTTGAGGATCAGCC | GGTGCCTGAGTTGAATCTC | Schaart et al. 2013 [15] | AF401220 |
| *FaANR* | GCTATCAGGTTCCATATCCATTACAC | TGAGCTCGGCAGACATCCT | Schaart et al. 2013 [15] | DQ664193 |
| *FaUFGT* | ATCGTGGCTTGACAAACAGAA | TGACCACAAGAATGGAACCCTA | Salvatierra et al. 2010 [71] | AY695816 |
| *FaANS* | ATCGTCATGCACATAGGCGACACC | CCTTGGGCGGCTCACAGAAAA | Salvatierra et al. 2010 [71] | AY695817 |
| *FaLAR* | GGTGATGGCACGGTTAAAGC | CTCCCACAGTGAAGCAAGTCC | Salvatierra et al. 2010 [71] | JX134096 |
| *FaGAPDH* | TCCATCACTGCCACCCAGAAGACTG | AGCAGGCAGAACCTTTCCGACAG | Preuß et al. 2014 [70] | AF421145 |

^1^ Gene names: *FaJMT*, jasmonic acid methyltransferase; *FaJAR1.2*, jasmonic acid-amide synthetase; *FaMYC2*, basic-helix-loop-helix transcription factor MYC 2; *FaJAZ1*, jasmonate-zim-domain 1; *FaMYB9*, MYB transcription factor 9; *FaMYB10*, MYB transcription factor 10; *FaMYB11*, MYB transcription factor 11; *FabHLH3*, basic-helix-loop-helix transcription factor 3; *FabHLH33*, basic-helix-loop-helix transcription factor 33; *FaMYB1* MYB transcription factor 1; *FaANR*, anthocyanidin reductase; *FaUFGT*, uridine diphosphate glucose-flavonoid glucosyltransferase; *FaANS*, anthocyanidin synthase; *FaLAR*, leucoanthocyanidin reductase; *GAPDH*, glyceraldehyde 3-phosphate dehydrogenase.

^2^ GenBank accession numbers of gene sequences used for primer design. In the case of *FaJMT, FaJAR1.2* and *FaMYC2* primer sequences were designed from full-length cDNA sequences of *Fragaria vesca*.
