# Supplementary material for: Application of a JA-Ile Biosynthesis Inhibitor to Methyl Jasmonate-Treated Strawberry Fruit Induces Upregulation of Specific MBW Complex-Related Genes and Accumulation of Proanthocyanidins
Source: Molecules. 2018 Jun 13;23(6):1433. doi: 10.3390/molecules23061433 (PMC6100305; doi:10.3390/molecules23061433)
Supplement: Supplementary file 1 [file molecules-23-01433-s001.zip › Table S10.docx]

**Table S10.** Genomic data and expression patterns during fruit development and ripening for anthocyanidin synthase (*ANS*), UDP glucose:flavonoid 3-O-glucosyltransferase (*UFGT*), anthocyanidin reductase (*ANR*), and leucoanthocyanidin reductase (*LAR*) gene family in *Fragaria vesca* and *Fragaria* x *ananassa*, respectively.

|  | ***Fragaria vesca*** | | | | | | | | | | ***Fragaria* x *ananassa*** | | | | | | | |
| --- | --- | --- | --- | --- | --- | --- | --- | --- | --- | --- | --- | --- | --- | --- | --- | --- | --- | --- |
| **Gene name ^1^** | **Predicted sequences ^2^** | **GeneID** | **Accession** | **Chromosome** | **Start** | **End** | **Gene (bp)** | **CDS (bp)** | **Protein (aa)** | **Gene accession ^3^** | **FPKM expression values ^4^** | | | | | | | |
|  |  |  |  |  |  |  |  |  |  |  | **Achene** | | | | **Receptacle** | | | |
|  |  |  |  |  |  |  |  |  |  |  | **GA** | **WA** | **TA** | **RA** | **GR** | **WR** | **TR** | **RR** |
| Anthocyanidin synthase (*ANS*) | Leucoanthocyanidin dioxygenase ^5^ | 101308284 | NC_020495 | 5 | 693146 | 694409 | 1604 | 1151 | 383 | XM_004298672.2 | 20.05 | 0.14 | 5.71 | 35.89 | 201.10 | 9.09 | 412.55 | 819.19 |
|  | Leucoanthocyanidin dioxygenase-like | 101314183 | NC_020495 | 5 | 11181226 | 11182623 | 1398 | 1067 | 355 | XM_004299584.2 | 102.22 | 20.26 | 51.32 | 38.57 | 115.86 | 3.52 | 2.54 | 7.52 |
|  | Leucoanthocyanidin dioxygenase-like | 101309875 | NC_020491 | 1 | 2453939 | 2457603 | 1716 | 1151 | 383 | XM_004288720.2 | 95.65 | 25.48 | 19.17 | 46.02 | 26.34 | 33.15 | 300.94 | 1113.03 |
| UDP glucose:flavonoid 3-O-glucosyltransferase (*UFGT*) | UDP-glucose flavonoid 3-O-glucosyltransferase 7-like | 101305722 | NC_020492 | 2 | 3456103 | 3459026 | 1953 | 1818 | 605 | XM_011461372.1 | 0.02 | 0.04 | 0.02 | 0.00 | 0.00 | 0.00 | 0.00 | 0.01 |
|  | Putative UDP-glucose flavonoid 3-O-glucosyltransferase 3 | 101293172 | NC_020493 | 3 | 4725190 | 4726620 | 1840 | 1430 | 476 | XM_011462108.1 | 1.74 | 0.49 | 1.62 | 1.88 | 0.28 | 0.81 | 4.37 | 4.03 |
|  | Putative UDP-glucose flavonoid 3-O-glucosyltransferase 3 | 101309070 | NC_020496 | 6 | 28042879 | 28044315 | 1937 | 1436 | 478 | XM_004303905.2 | 0.18 | 0.78 | 2.84 | 9.74 | 0.06 | 0.01 | 0.17 | 2.46 |
|  | Putative UDP-glucose flavonoid 3-O-glucosyltransferase 3 | 101309367 | NC_020496 | 6 | 28045389 | 28046936 | 2031 | 1547 | 515 | XM_004303906.2/XM_004303907.2 | 13.32 | 4.10 | 6.39 | 10.28 | 0.60 | 2.68 | 23.24 | 24.52 |
|  | UDP-glucose flavonoid 3-O-glucosyltransferase 6-like | 101309653 | NC_020496 | 6 | 28049051 | 28050478 | 1856 | 1427 | 475 |  |  |  |  |  |  |  |  |  |
|  | UDP-glucose flavonoid 3-O-glucosyltransferase 6 | 101310433 | NC_020496 | 6 | 28063756 | 28065195 | 2084 | 1439 | 479 | XM_004303908.2 | 31.12 | 49.72 | 166.08 | 209.68 | 1.53 | 1.06 | 1.59 | 2.38 |
|  | UDP-glucose flavonoid 3-O-glucosyltransferase 7 | 101304787 | NC_020496 | 6 | 28673573 | 28675036 | 1791 | 1463 | 487 | XM_004303973.2 | 20.47 | 13.12 | 16.65 | 18.59 | 11.34 | 15.58 | 26.97 | 34.85 |
|  | UDP-glucose flavonoid 3-O-glucosyltransferase 7-like | 105352714 | NC_020496 | 6 | 28686383 | 28687861 | 2771 | 1338 | 445 | XM_011470321.1 | 0.38 | 0.59 | 0.21 | 0.49 | 0.13 | 0.16 | 0.25 | 0.12 |
|  | UDP-glucose flavonoid 3-O-glucosyltransferase 7-like | 101301127 | NC_020496 | 6 | 28692095 | 28693732 | 1973 | 1511 | 503 | XM_004305891.2 | 0.00 | 0.00 | 0.00 | 0.00 | 0.00 | 0.00 | 0.00 | 0.00 |
|  | UDP-glucose flavonoid 3-O-glucosyltransferase 7-like | 101305962 | NC_020496 | 6 | 28703122 | 28704534 | 1920 | 1412 | 470 | XM_011469430.1 | 22.50 | 1.94 | 1.13 | 1.81 | 23.49 | 10.35 | 9.21 | 4.92 |
|  | UDP-glucose flavonoid 3-O-glucosyltransferase 7-like | 101306252 | NC_020496 | 6 | 28706266 | 28707717 | 2092 | 1451 | 483 | XM_004303976.2 | 0.09 | 0.06 | 0.62 | 0.64 | 1.22 | 4.27 | 8.71 | 7.35 |
|  | UDP-glucose flavonoid 3-O-glucosyltransferase 7-like | 101306543 | NC_020496 | 6 | 28709038 | 28710492 | 1875 | 1454 | 484 | XM_004303977.2 | 7.69 | 11.06 | 19.96 | 21.74 | 22.78 | 57.25 | 155.45 | 140.45 |
|  | UDP-glucose flavonoid 3-O-glucosyltransferase 7-like | 101301417 | NC_020496 | 6 | 28712499 | 28713956 | 1972 | 1457 | 485 | XM_004305892.2 | 0.02 | 0.00 | 0.00 | 0.00 | 0.02 | 0.00 | 0.00 | 0.00 |
|  | UDP-glucose flavonoid 3-O-glucosyltransferase 7-like | 101301997 | NC_020496 | 6 | 28719681 | 28721105 | 1767 | 1424 | 474 | XM_004305893.2 | 1.36 | 0.45 | 0.55 | 0.51 | 1.19 | 2.06 | 4.55 | 1.85 |
|  | Putative UDP-glucose flavonoid 3-O-glucosyltransferase 3 | 101312851 | NC_020496 | 6 | 36061096 | 36062562 | 1756 | 1466 | 488 | XM_004304464.2 | 1.88 | 19.22 | 11.71 | 10.71 | 0.22 | 0.18 | 0.24 | 0.11 |
|  | UDP-glucose flavonoid 3-O-glucosyltransferase 7-like | 101296902 | NC_020497 | 7 | 16125204 | 16126607 | 1404 | 1404 | 467 | XM_011472302.1 | 3.51 | 5.78 | 8.51 | 8.83 | 0.56 | 1.08 | 2.15 | 2.90 |
|  | Putative UDP-glucose flavonoid 3-O-glucosyltransferase 3 | 101308142 | NW_004440700 | Unknown | 1103 | 2500 | 1899 | 1397 | 465 | XM_004309252.2 | N/A | N/A | N/A | N/A | N/A | N/A | N/A | N/A |
|  | Anthocyanidin 3-O-glucosyltransferase 2-like isoform X1 | 101313438 | NC_020496 | 6 | 36092614 | 36094666 | 1918 | 1496 | 498 | XM_004304466.2/XM_011469878.1 | 0.25 | 0.10 | 0.03 | 0.05 | 0.06 | 0.07 | 0.28 | 0.39 |
|  | Anthocyanidin 3-O-glucosyltransferase 2-like isoform X2 | 101313438 | NC_020496 | 6 | 36092614 | 36094666 | 1846 | 1424 | 474 |  |  |  |  |  |  |  |  |  |
|  | Anthocyanidin 3-O-glucosyltransferase 2-like transcript variant X1 | 101313142 | NC_020496 | 6 | 36089385 | 36091745 | 2113 | 1427 | 475 | XM_011469875.1 /XM_011469876.1 | 18.03 | 5.68 | 16.83 | 31.63 | 27.14 | 67.83 | 150.99 | 153.48 |
|  | Anthocyanidin 3-O-glucosyltransferase 2-like transcript variant X2 | 101313142 | NC_020496 | 6 | 36089385 | 36091745 | 2027 | 1427 | 475 |  |  |  |  |  |  |  |  |  |
|  | Anthocyanidin 3-O-glucosyltransferase 7-like | 101314250 | NC_020492 | 2 | 2242950 | 2244804 | 1769 | 1364 | 454 | XM_004292023.2 | 0.00 | 0.00 | 0.00 | 0.00 | 0.02 | 0.00 | 0.00 | 0.00 |
|  | Anthocyanidin 3-O-glucosyltransferase 5-like | 101312164 | NC_020495 | 5 | 26732385 | 26733827 | 1443 | 1443 | 480 | XM_004301839.1 | 19.48 | 7.65 | 5.92 | 1.32 | 33.02 | 86.53 | 82.18 | 29.76 |
|  | Anthocyanidin 3-O-glucosyltransferase 5-like | 101311874 | NC_020495 | 5 | 26722318 | 26725224 | 2907 | 1475 | 491 | XM_004301838.2 | 1.60 | 25.75 | 15.03 | 21.58 | 1.02 | 1.25 | 0.59 | 1.45 |
|  | Anthocyanidin 3-O-glucosyltransferase 7-like | 101311844 | NC_020493 | 3 | 6046121 | 6047973 | 1767 | 1364 | 454 | XM_004295467.2 | 0.00 | 0.00 | 0.00 | 0.00 | 0.02 | 0.00 | 0.00 | 0.00 |
|  | Anthocyanidin 3-O-glucosyltransferase 5-like | 101311585 | NC_020495 | 5 | 26718349 | 26719791 | 1443 | 1443 | 480 | XM_004301837.1 | 0.05 | 0.00 | 0.00 | 0.03 | 0.00 | 0.01 | 0.00 | 0.00 |
|  | Anthocyanidin 3-O-glucosyltransferase 2-like | 101311262 | NC_020493 | 3 | 6027272 | 6029349 | 2078 | 1421 | 473 | XM_004295466.2 | 0.22 | 0.02 | 0.18 | 0.05 | 0.09 | 0.02 | 0.02 | 0.00 |
|  | Anthocyanidin 3-O-glucosyltransferase 5-like | 101311007 | NC_020495 | 5 | 26707226 | 26708978 | 1753 | 1487 | 495 | XM_004301835.2 | 0.00 | 0.00 | 0.00 | 0.00 | 0.00 | 0.00 | 0.01 | 0.00 |
|  | Anthocyanidin 3-O-glucosyltransferase 5-like | 101310867 | NC_020492 | 2 | 32636671 | 32638852 | 2182 | 1439 | 479 | XM_004293173.2 | 2.66 | 2.05 | 6.45 | 4.36 | 4.35 | 5.83 | 7.99 | 6.36 |
|  | Anthocyanidin 5,3-O-glucosyltransferase | 101302986 | NC_020497 | 7 | 22041743 | 22043843 | 2101 | 1415 | 471 | XM_004307988.2 | 0.04 | 0.02 | 0.00 | 0.00 | 0.01 | 0.00 | 0.00 | 0.00 |
|  | Anthocyanidin 3-O-glucosyltransferase 2 ^6^ | 101300000 | NC_020497 | 7 | 20360656 | 20362545 | 1724 | 1397 | 465 | XM_004307828.2 | 0.64 | 0.57 | 2.80 | 40.90 | 0.24 | 4.20 | 725.14 | 843.95 |
|  | Anthocyanidin 3-O-glucosyltransferase 5-like | 101299171 | NC_020493 | 3 | 20319885 | 20321274 | 1390 | 959 | 319 | XM_004295959.2 | 0.00 | 0.00 | 0.00 | 0.00 | 0.02 | 0.01 | 0.00 | 0.00 |
|  | Anthocyanidin 5,3-O-glucosyltransferase-like | 101298317 | NC_020495 | 5 | 14540939 | 14542910 | 1972 | 1436 | 478 | XM_004299770.2 | 5.83 | 1.34 | 0.51 | 0.35 | 0.63 | 6.03 | 14.36 | 8.61 |
|  | Anthocyanidin 3-O-glucosyltransferase 5-like | 101296671 | NC_020495 | 5 | 26699604 | 26701621 | 1878 | 1451 | 483 | XM_004300555.2 | 0.07 | 0.00 | 0.00 | 0.00 | 0.16 | 0.28 | 0.17 | 0.00 |
|  | Anthocyanidin 3-O-glucosyltransferase 2-like | 101295404 | NC_020493 | 3 | 12693578 | 12695513 | 1936 | 1436 | 478 | XM_004294212.2 | 14.77 | 9.74 | 8.05 | 8.47 | 15.93 | 5.63 | 7.55 | 13.86 |
|  | Anthocyanidin 3-O-glucosyltransferase 7-like | 101291082 | NC_020491 | 1 | 3543582 | 3546229 | 1952 | 1388 | 462 | XM_011460202.1 | 58.03 | 2.24 | 3.17 | 1.15 | 1.52 | 0.08 | 0.07 | 0.08 |
|  | Anthocyanidin 3-O-glucosyltransferase 2-like | 105352450 | NC_020496 | 6 | 31421872 | 31423442 | 1571 | 1388 | 462 | XM_011469617.1 | 5.27 | 4.63 | 11.90 | 17.10 | 1.85 | 59.43 | 228.70 | 253.81 |
| Anthocyanidin reductase (*ANR*) | Anthocyanidin reductase-like isoform X1 | 101307502 | NC_020495 | 5 | 2486162 | 2488144 | 1982 | 1037 | 345 | XM_004300815.2, XM_011465671.1, XM_011465672.1 | 0.40 | 0.29 | 0.60 | 0.44 | 0.23 | 9.60 | 12.70 | 4.17 |
|  | Anthocyanidin reductase-like isoform X2 | 101307502 | NC_020495 | 5 | 2486162 | 2488144 | 1547 | 992 | 330 |  |  |  |  |  |  |  |  |  |
|  | Anthocyanidin reductase-like isoform X3 | 101307502 | NC_020495 | 5 | 2486162 | 2488144 | 1155 | 890 | 296 |  |  |  |  |  |  |  |  |  |
|  | Anthocyanidin reductase-like | 101295450 | NC_020497 | 7 | 3850597 | 3852977 | 1617 | 1079 | 359 | XM_004306642.2 | 1.06 | 2.57 | 7.26 | 12.54 | 0.54 | 0.27 | 0.17 | 0.11 |
|  | Anthocyanidin reductase ^7^ | 101297977 | NW_004443416 | Unknown | 66746 | 68889 | 1389 | 1025 | 341 | XM_004309614.2 | 284.95 | 67.78 | 35.37 | 42.73 | 635.30 | 49.29 | 16.53 | 6.64 |
| Leucoanthocyanidin reductase (*LAR*) | Leucoanthocyanidin reductase-like ^8^ | 101306809 | NC_020494 | 4 | 16322523 | 16325200 | 1574 | 1052 | 350 | XM_004297096.2 | 35.06 | 2.23 | 3.36 | 2.70 | 72.75 | 13.38 | 20.83 | 22.32 |

^1^ Gene name refers to *F.* x *ananassa* nomenclature assigned in this research.

^2^ Names of predicted sequences obtained from the *F. vesca* genome in National Center for Biotechnology Information (NCBI, <https://www.ncbi.nlm.nih.gov/genome>).

^3^ Gene accession for predicted sequences in *F. vesca* genome used in Sánchez-Sevilla et al. [52] for RNAseq assays in *F*. x *ananassa*.

^4^ Original fragments per kilobase of exon per million fragments (FPKM) values of *ANS*, *UFGT*, *ANR* and *LAR* genes in achene and receptacle during development and ripening of *F*. x *ananassa* fruits [52]. GA, green achene; RA, red achene; TA, turning achene; WA, white achene; GR, green receptacle; RR, red receptacle; TR, turning receptacle; WR, white receptacle.

^5^ Predicted sequence in *F. vesca* genome corresponding to the *F.* x *ananassa* *ANS* analyzed in the present research. This sequence corresponds to the previously reported by Almeida et al. [13] (GenBank AY695818 and AY695817) and in NCBI (GenBank JX134095 and JQ923457).

^6^ Predicted sequence in *F. vesca* genome corresponding to the *F.* x *ananassa* *UFGT* analyzed in the present research. This sequence corresponds to the previously reported by Almeida et al. [13] (GenBank AY695816 and AY695815), Lunkenbein et al. [72] (GenBank AY663784) and in NCBI (GenBank AY575056).

^7^ Predicted sequence in *F. vesca* genome corresponding to the *F.* x *ananassa* *ANR* analyzed in the present research. This sequence corresponds to the previously reported by Almeida et al. [13] (GenBank DQ664193 and DQ664192) and in NCBI (GenBank DQ438979 and JX271492).

^8^ Predicted sequence in *F. vesca* genome corresponding to the *F.* x *ananassa* *LAR* analyzed in the present research. This sequence corresponds to the previously reported by Almeida et al. [13] (GenBank DQ087253 and DQ834906) and in NCBI (GenBank JX134096).
