# Supplementary material for: Application of a JA-Ile Biosynthesis Inhibitor to Methyl Jasmonate-Treated Strawberry Fruit Induces Upregulation of Specific MBW Complex-Related Genes and Accumulation of Proanthocyanidins
Source: Molecules. 2018 Jun 13;23(6):1433. doi: 10.3390/molecules23061433 (PMC6100305; doi:10.3390/molecules23061433)
Supplement: Supplementary file 1 [file molecules-23-01433-s001.zip › Table S2.docx]

**Table S2.** Changes (Δ) in fruit firmness (N) and weight (g) at different treatments during the *in vitro* ripening of strawberry fruits.

| **Treatment ^1^** | **Δ Firmness (N)** | | | **Δ Weight (g)** | | |
| --- | --- | --- | --- | --- | --- | --- |
|  | **0 h** | | | **0 h** | | |
| **Untreated** | 6.32 ± 0.96 | | | 3.56 ± 0.96 | | |
| **Treated** | **12 h** | **24 h** | **48 h** | **12 h** | **24 h** | **48 h** |
| MeJA | -0.19 ± 0.34b ^2^ | -0.17 ± 0.17a | 0.35 ± 0.36a | 1.72 ± 0.41a* | 0.02 ± 0.35a | 1.07 ± 0.58b |
|  | (2.71 - 2.89) | (2.46 - 2.62) | (2.10 - 1.75) | (5.65 - 3.93) | (5.50 - 5.48) | (5.41 - 4.34) |
| jarin-1 | -1.03 ± 0.19a* | -0.77 ± 0.11a* | -0.03 ± 0.16a | 0.77 ± 0.34a | 1.78 ± 0.44b* | 0.05 ± 0.25ab |
|  | (2.24 - 3.27) | (1.92 - 2.70) | (2.01 - 2.04) | (6.72 - 5.94) | (6.74 - 4.96) | (5.38 - 5.33) |
| MeJA+jarin-1 | --- | --- | 0.28 ± 0.18a | --- | --- | -0.44 ± 0.31a |
|  |  |  | (2.18 - 1.89) |  |  | (4.39 - 4.83) |

^1^ MeJA and jarin-1 treatments involved the application of 100 μM MeJA and 60 μM jarin-1, and measurements were performed at 12, 24, and 48 h. MeJA+jarin-1 treatment involved the addition of 60 μM jarin-1 to 100 μM MeJA solution at 24 h and measurements were performed at 48 h. For details, see Scheme 1.

^2^ Values (delta, Δ) are mean of three biological replicates ± S.E normalized. Delta was calculated as the difference between the mean of treatments and their respective controls at each time (Treatment – Control). Lowercase letters correspond to significant differences between treatments at the same time. Asterisks indicate significant differences with each control treatment. Differences were considered statistically significant at p≥0.05 (LSD test).
