# Supplementary material for: Application of a JA-Ile Biosynthesis Inhibitor to Methyl Jasmonate-Treated Strawberry Fruit Induces Upregulation of Specific MBW Complex-Related Genes and Accumulation of Proanthocyanidins
Source: Molecules. 2018 Jun 13;23(6):1433. doi: 10.3390/molecules23061433 (PMC6100305; doi:10.3390/molecules23061433)
Supplement: Supplementary file 1 [file molecules-23-01433-s001.zip › Table S13.docx]

**Table S13.** Primer sequences used for promoter isolation of *MYB* genes and primer walking sequencing.

| **Gene** | **Forward (5´→3´)** | **Reverse (5´→3´)** | **Amplicon size (bp)** |
| --- | --- | --- | --- |
| *FaMYB1* | ACGAGGTGAGAACTCTGTGC | CCGTTGGGGGAGAATCTGG | 1996 |
| *FaMYB9* | GCCAACATTAACTTGCCGGA | CCAACTGGGTTTGAGGAACTTG | 2808 |
| *FaMYB10* | TTCCGCATTGCAGTCGACAA | CATGCACCTTTTCTCACACCG | 2080 |
| *FaMYB11* | GACCGAAGGACCAAACTGATG | GGTACCAGAAGCTATCGGCC | 2395 |
