# Supplementary material for: Application of a JA-Ile Biosynthesis Inhibitor to Methyl Jasmonate-Treated Strawberry Fruit Induces Upregulation of Specific MBW Complex-Related Genes and Accumulation of Proanthocyanidins
Source: Molecules. 2018 Jun 13;23(6):1433. doi: 10.3390/molecules23061433 (PMC6100305; doi:10.3390/molecules23061433)
Supplement: Supplementary file 1 [file molecules-23-01433-s001.zip › Table S1.docx]

**Table S1.** Changes (Δ) in fruit skin color according to CIEL*a*b* scale, chroma, and hue at different time points under treatments during the *in vitro* ripening of strawberry fruits.

| **Time** | **Treatment ^1^** | **Color parameters** | | | | |
| --- | --- | --- | --- | --- | --- | --- |
|  |  | **ΔL*** | **Δa*** | **Δb*** | **ΔChroma** | **ΔHue (h°)** |
| 0 h | Untreated | 55.17 ± 3.69 | -9.6 ± 2.16 | 35.18 ± 3.37 | 36.5 ± 3.66 | 254.83 ± 2.58 |
| 12 h | MeJA | 0.21 ± 1.19a ^2^ | 1.58 ± 1.19a | -0.89 ± 1.02a | -1.12 ± 1.14a | -2.69 ± 2.01a |
|  |  | (57.62 - 57.41) | (-7.62 - -9.23) | (32.25 - 33.14) | (33.33 - 34.45) | (102.74 - 105.44) |
|  | jarin-1 | 4.33 ± 0.54b* | -0.04 ± 0.52a | -1.58 ± 0.67a | -1.53 ± 0.78a | 0.76 ± 0.60a |
|  |  | (59.18 - 54.85) | (-9.11 - -9.07) | 31.72 - 33.30 | (33.02 - 34.55) | (105.89 - 105.14) |
| 24 h | MeJA | -2.7 ± 1.27a | 2.89 ± 2.31a | 1.24 ± 0.6b | 1.3 ± 0.8a | -4.85 ± 3.45a |
|  |  | (55.26 - 57.96) | (-6.29 - -9.18) | (34.82 - 33.57) | (36.15 - 34.86) | (100.23 - 105.08) |
|  | jarin-1 | 2.26±1.37b | 2.33±0.58a | -1.54±0.6a | -2.05±0.62b* | -3.19 ± 0.86a |
|  |  | (58.50 - 56.24) | (-7.18 - -9.50) | (32.75 - 34.29) | (33.57 - 35.62) | (102.25 - 105.44) |
| 48 h | MeJA | -3.3 ± 1.90a | 7.23 ± 4.69b | -0.78 ± 0.92a | -0.47 ± 1.33a | -12.3 ± 7.01a |
|  |  | (47.67 - 50.97) | (5.70 - -1.54) | (34.36 - 35.14) | (38.01 - 38.48) | (82.09 - 94.34) |
|  | jarin-1 | 2.16 ± 0.91b | -3.53 ± 0a | 0.44 ± 0.62a | -1.01 ± 0.66a | 4.81 ± 1.42b |
|  |  | (53.96 - 51.80) | (-6.45 - -2.92) | (35.34 - 34.90) | (36.04 - 37.05) | (100.26 - 95.44) |
|  | MeJA+jarin-1 | -0.04 ± 2.10ab | -1.6 ± 4.17a | 0.89 ± 0.78a | 1.57 ± 0.85a | 1.78 ± 6.2b |
|  |  | (51.76 - 51.39) | (-4.53 - -2.23) | (35.80 - 35.02) | (38.62 - 37.76) | (97.22 - 94.89) |

^1^ MeJA and jarin-1 treatments involved the application of 100 μM MeJA and 60 μM jarin-1, and measurements were performed at 12, 24, and 48 h. MeJA+jarin-1 treatment involved the addition of 60 μM jarin-1 to 100 μM MeJA solution at 24 h and measurements were performed at 48 h. For details, see Scheme 1.

^2^ Values (delta, Δ) are mean of three biological replicates ± S.E normalized. Delta was calculated as the difference between the mean of treatments and their respective controls at each time (Treatment – Control). Lowercase letters correspond to significant differences between treatments at the same time. Asterisks indicate significant differences with each control treatment. Differences were considered statistically significant at p≥0.05 (LSD test).
