# Supplementary material for: Application of a JA-Ile Biosynthesis Inhibitor to Methyl Jasmonate-Treated Strawberry Fruit Induces Upregulation of Specific MBW Complex-Related Genes and Accumulation of Proanthocyanidins
Source: Molecules. 2018 Jun 13;23(6):1433. doi: 10.3390/molecules23061433 (PMC6100305; doi:10.3390/molecules23061433)
Supplement: Supplementary file 1 [file molecules-23-01433-s001.zip › Table S12.docx]

**Table S12.** Predicted sequences of *Fragaria* x *ananassa* *MYB* genes on *Fragaria vesca* genome.

| ***Fragaria* x *ananassa* ^1^** | | **Genome BLAST analysis** | | | | ***Fragaria vesca*** | | |
| --- | --- | --- | --- | --- | --- | --- | --- | --- |
| **Gene ^2^** | **GenBank** | **Total score** | **Query cover (%)** | **E-value** | **Identity (%)** | **Predicted mRNA** | **Gene ID** | **Gene name ^2^** |
| *FaMYB1* | AF401220 | 1327 | 87% | 0.0 | 88% | Transcription factor MYB3-like | 101311479 | *FvMYB1* |
| *FaMYB9* | JQ989281 | 1782 | 98% | 0.0 | 97% | Transcription factor TT2-like | 101301599 | *FvMYB9* |
| *FaMYB10* | MG456859 | 1140 | 100% | 0.0 | 95 % | Transcription factor MYB114-like | 101307024 | *FvMYB10* |
| *FaMYB11* | JQ989282 | 1936 | 100% | 0.0 | 97% | Transcription factor TT2-like | 101296688 | *FvMYB11* |

^1^ *Fragaria* x *ananassa* sequences used as query and genome database of *Fragaria vesca* as subject.

^2^ This refers to the gene names assigned in the present research.
