# Supplementary material for: Application of a JA-Ile Biosynthesis Inhibitor to Methyl Jasmonate-Treated Strawberry Fruit Induces Upregulation of Specific MBW Complex-Related Genes and Accumulation of Proanthocyanidins
Source: Molecules. 2018 Jun 13;23(6):1433. doi: 10.3390/molecules23061433 (PMC6100305; doi:10.3390/molecules23061433)
Supplement: Supplementary file 1 [file molecules-23-01433-s001.zip › Table S7.docx]

**Table S7.** Changes (Δ) in relative expression levels of jasmonate pathway-related genes at different treatments during the *in vitro* ripening of strawberry fruits.

| **Time** | **Treatment ^1^** | Δ **Relative expression** | | | |
| --- | --- | --- | --- | --- | --- |
|  |  | ***FaJMT*** | ***FaJAR1.2*** | ***FaMYC2*** | ***FaJAZ1*** |
| 12 h | MeJA | -0.62 ± 0.47a ^2^ | 1.48 ± 0.30a* | -0.24 ± 0.19a | -0.26 ± 0.11a |
|  |  | (1.39 - 2.01) | (2.96 - 1.48) | (0.88 - 1-13) | (0.36 - 0.62) |
|  | jarin-1 | 0.45 ± 0.51a | -0.24 ± 0.74a | -0.22 ± 0.13a | -0.48 ± 0.25a |
|  |  | (1.67 - 1.22) | (2.36 - 2.59) | (0.59 - 0.82) | (0.33 - 0.81) |
| 24 h | MeJA | -0.87 ± 0.31a | -1.99 ± 0.92a | -0.74 ± 0.26a | -0.10 ± 0.23a |
|  |  | (0.31 - 1.17) | (1.38 - 3.37) | (0.93 - 1.67) | (0.20 - 0.30) |
|  | jarin-1 | -0.56 ± 0.43a | -1.26 ± 1.54a | 0.09 ± 0.07b* | -0.06 ± 0.03a |
|  |  | (0.59 - 1.15) | (1.53 - 2.79) | (0.68 - 0.60) | (0.12 - 0.18) |
| 48 h | MeJA | -0.52 ± 0.20a | -0.66 ± 0.16a | -1.47 ± 0.16a* | -0.04 ± 0.14a |
|  |  | (0.54 - 1.06) | (1.15 - 1.81) | (0.62 - 2.09) | (0.14 - 0.17) |
|  | jarin-1 | 2.54 ± 0.74a | 4.13 ± 4.23a | -0.21 ± 0.08c | 0.07 ± 0.09a |
|  |  | (3.42 - 0.89) | (5.56 - 1.43) | (0.44 - 0.65) | (0.17 - 0.10) |
|  | MeJA+jarin-1 ^3^ | 29.38 ± 7.03b* | 82.05 ± 38.33b* | -0.65 ± 0.01b* | 0.21 ± 0.08a* |
|  |  | (30.355 – 0.975) | (84.12 – 1.62) | (0.72 - 1.37) | (0.35 - 0.14) |

^1^ MeJA and jarin-1 treatments involved the application of 100 μM MeJA and 60 μM jarin-1, and measurements were performed at 12, 24, and 48 h. MeJA+jarin-1 treatment involved the addition of 60 μM jarin-1 to 100 μM MeJA solution at 24 h and the measurements were performed at 48 h. For details, see Scheme 1.

^2^ Values (delta, Δ) are mean of three biological replicates ± S.E normalized. Delta was calculated as the difference between the mean of treatments and their respective controls at each time (Treatment – Control). Lowercase letters correspond to significant differences between treatments at the same time. Asterisks indicate significant differences with each control treatment. Differences were considered statistically significant at p≥0.05 (LSD test).
