# Supplementary material for: Application of a JA-Ile Biosynthesis Inhibitor to Methyl Jasmonate-Treated Strawberry Fruit Induces Upregulation of Specific MBW Complex-Related Genes and Accumulation of Proanthocyanidins
Source: Molecules. 2018 Jun 13;23(6):1433. doi: 10.3390/molecules23061433 (PMC6100305; doi:10.3390/molecules23061433)
Supplement: Supplementary file 1 [file molecules-23-01433-s001.zip › Table S6.docx]

**Table S6.** Changes in relative expression levels of MBW complex-related genes at different treatments during the *in vitro* ripening of strawberry fruits.

| **Time** | **Treatment ^1^** | **Δ Relative expression** | | | | | |
| --- | --- | --- | --- | --- | --- | --- | --- |
|  |  | ***FaMYB9*** | ***FaMYB11*** | ***FabHLH33*** | ***FaMYB1*** | ***FaMYB10*** | ***FabHLH3*** |
| 12 h | MeJA | 0.27 ± 0.37a ^2^ | 0.36 ± 0.94a | 0.08 ± 0.82a | -0.26 ± 0.73a | 9.91 ± 18.61a | 6.72 ± 1.01b* |
|  |  | (0.80 - 0.53) | (1.74 - 1.38) | (1.49 - 1.41) | (1.67 - 1.92) | (11.41 - 1.50) | (8.12 - 1.41) |
|  | jarin-1 | -0.10 ± 0.27a | -0.44 ± 0.49a | -0.28 ± 0.28a | -0.88 ± 0.15a | 28.49 ± 140.38a | -0.09 ± 0.16a |
|  |  | (0.62 - 0.72) | (0.94 - 1.37) | (0.78 - 1.06) | (1.14 - 2.02) | (204.43 - 175.94) | (1.12 - 1.21) |
| 24 h | MeJA | -0.39 ± 0.32a | 0.24 ± 1.12a | -0.13 ± 0.23a | 0.01 ± 0.04a | 275.22 ± 508.86a | 0.22 ± 0.41a |
|  |  | (0.24 - 0.63) | (0.71 - 0.48) | (0.44 - 0.57) | (1.53 - 1.52) | (1069.61 - 794.39) | (1.72 - 1.50) |
|  | jarin-1 | -0.14 ± 0.27a | -0.41 ± 0.54a | 0.03 ± 0.16a | -0.65 ± 0.07a | 178.24 ± 98.88a | -0.24 ± 0.10a |
|  |  | (0.22 - 0.36) | (0.35 – 0.77) | (0.69 - 0.66) | (0.82 - 1.46) | (290.94 - 112.70) | (1.29 - 1.53) |
| 48 h | MeJA | -0.28 ± 0.10a | -0.22 ± 0.62a | -0.11 ± 0.11a | 1.63 ± 0.22b* | 366.73 ± 822.40a | -0.19 ± 0.01a |
|  |  | (0.19 - 0.47) | (0.65 - 0.86) | (0.39 - 0.50) | (2.24 - 0.50) | (1742.55 - 1375.82) | (1.34 - 1.53) |
|  | jarin-1 | 0.87 ± 1.09a | 0.99 ± 1.34a | 0.65 ± 0.90a | 0.56 ± 0.49a | 325.62 ± 989.24a | -0.45 ± 0.26a |
|  |  | (1.59 - 0.71) | (2.11 - 1.12) | (1.03 - 0.38) | (1.37 - 0.81) | (1681.12 - 1355.50) | (0.75 - 1.20) |
|  | MeJA+jarin-1 | 12.79 ± 3.4b* | 27.96 ± 7.60b* | 3.03 ± 0.79b* | 3.08 ± 0.91c* | -211.43 ± 306.57a | -0.17 ± 0.23a |
|  |  | (13.38 – 0.59) | (28.95 – 0.99) | (3.47 – 0.44) | (3.73 - 0.66) | (1154.23 - 1365.66) | (1.195 – 1.365) |

^1^ MeJA and jarin-1 treatments involved the application of 100 μM MeJA and 60 μM jarin-1, and measurements were performed at 12, 24, and 48 h. MeJA+jarin-1 treatment involved the addition of 60 μM jarin-1 to 100 μM MeJA solution at 24 h and the measurements were performed at 48 h. For details, see Scheme 1.

^2^ Values (delta, Δ) are mean of three biological replicates ± S.E normalized. Delta was calculated as the difference between the mean of treatments and their respective controls at each time (Treatment – Control). Lowercase letters correspond to significant differences between treatments at the same time. Asterisks indicate significant differences with each control treatment. Differences were considered statistically significant at p≥0.05 (LSD test).
