# Supplementary material for: Application of a JA-Ile Biosynthesis Inhibitor to Methyl Jasmonate-Treated Strawberry Fruit Induces Upregulation of Specific MBW Complex-Related Genes and Accumulation of Proanthocyanidins
Source: Molecules. 2018 Jun 13;23(6):1433. doi: 10.3390/molecules23061433 (PMC6100305; doi:10.3390/molecules23061433)
Supplement: Supplementary file 1 [file molecules-23-01433-s001.zip › Table S14.docx]

**Table S14.** PCR conditions for amplification of *MYB* promoters.

| **Cycle step** | ***FaMYB1*** | | ***FaMYB9*** | | ***FaMYB10*** | | ***FaMYB11*** | | **Cycles** |
| --- | --- | --- | --- | --- | --- | --- | --- | --- | --- |
|  | **Temp.** | **Time** | **Temp.** | **Time** | **Temp.** | **Time** | **Temp.** | **Time** |  |
| Initial Denaturation | 98 °C | 40 s | 98 °C | 40 s | 98 °C | 40 s | 98 °C | 40 s | 1 |
| Denaturation | 98 °C | 10 s | 98 °C | 10 s | 98 °C | 10 s | 98 °C | 10 s | 34 |
| Annealing | 62.6 °C | 30 s | 66.9 °C | 30 s | 68 °C | 30 s | 65 °C | 30 s |  |
| Extension | 72 °C | 63 s | 72 °C | 86 s | 72 °C | 68 s | 72 °C | 72 s |  |
| Final extension | 72 °C | 5 min | 72 °C | 5 min | 72 °C | 5 min | 72 °C | 5 min | 1 |
