# Supplementary figures and images for: Application of a JA-Ile Biosynthesis Inhibitor to Methyl Jasmonate-Treated Strawberry Fruit Induces Upregulation of Specific MBW Complex-Related Genes and Accumulation of Proanthocyanidins
Source: Molecules. 2018 Jun 13;23(6):1433. doi: 10.3390/molecules23061433 (PMC6100305; doi:10.3390/molecules23061433)

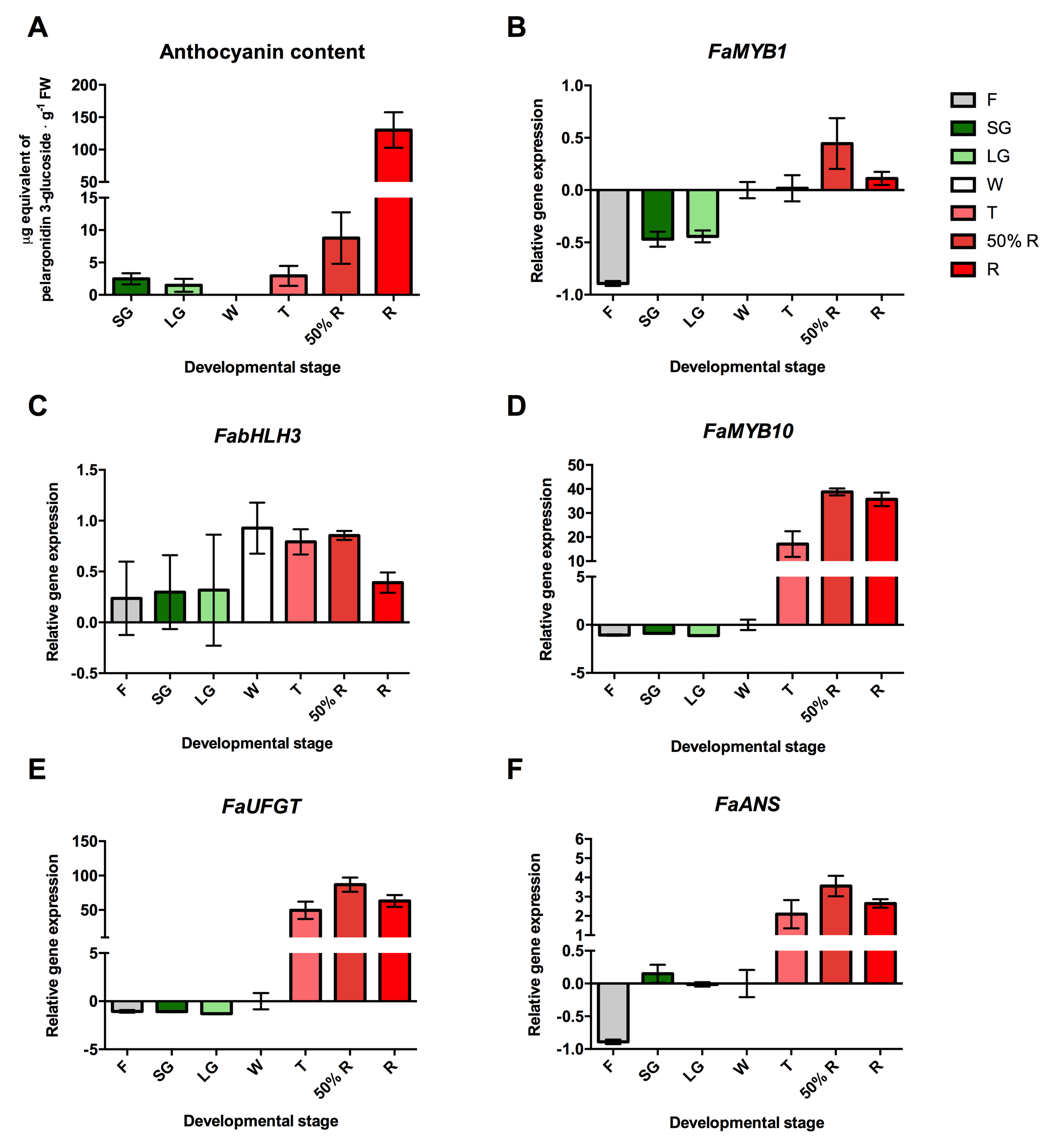

Supplement: Supplementary file 1 [file molecules-23-01433-s001.zip › Figure S3.tiff]

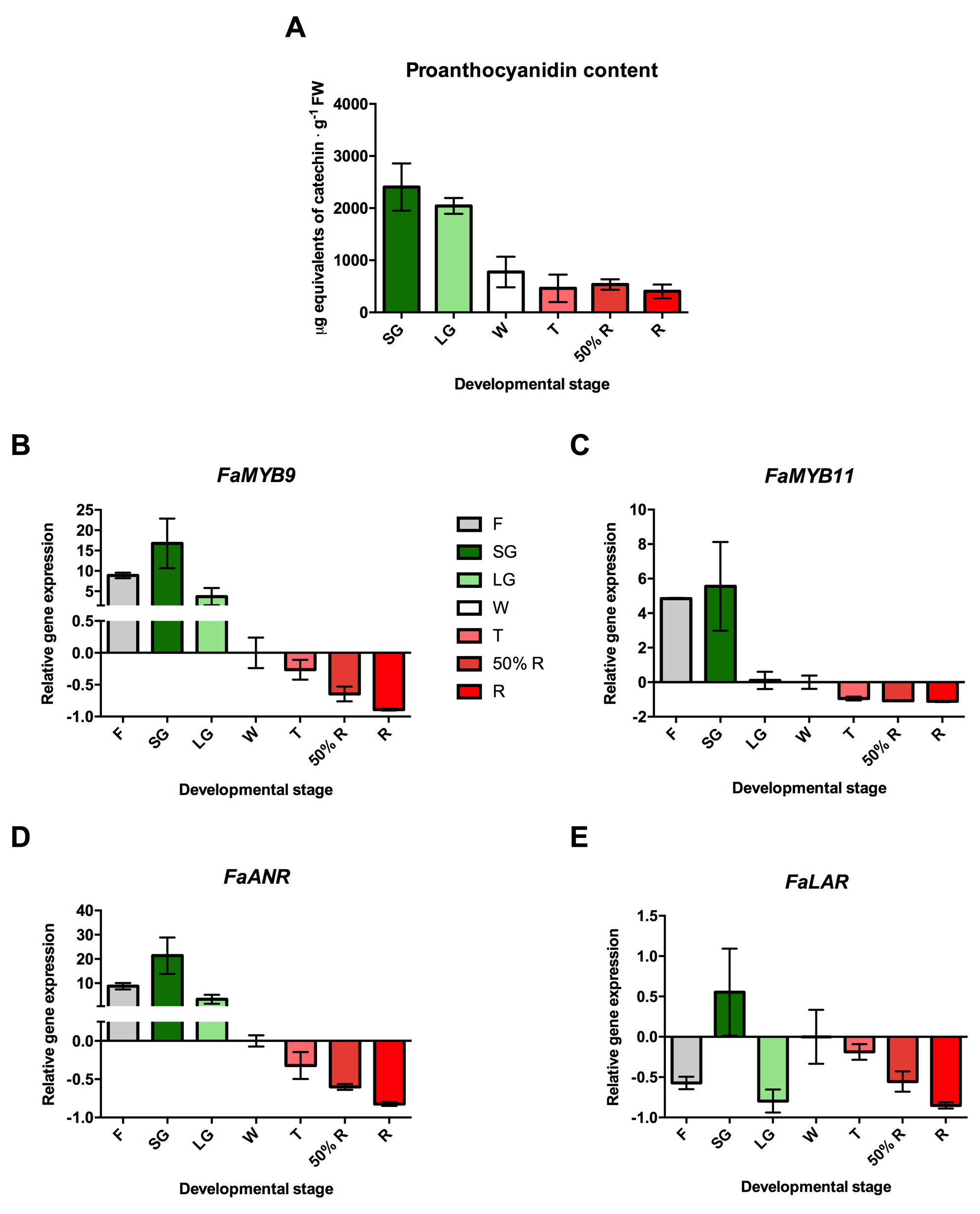

Supplement: Supplementary file 1 [file molecules-23-01433-s001.zip › Figure S2.tiff]

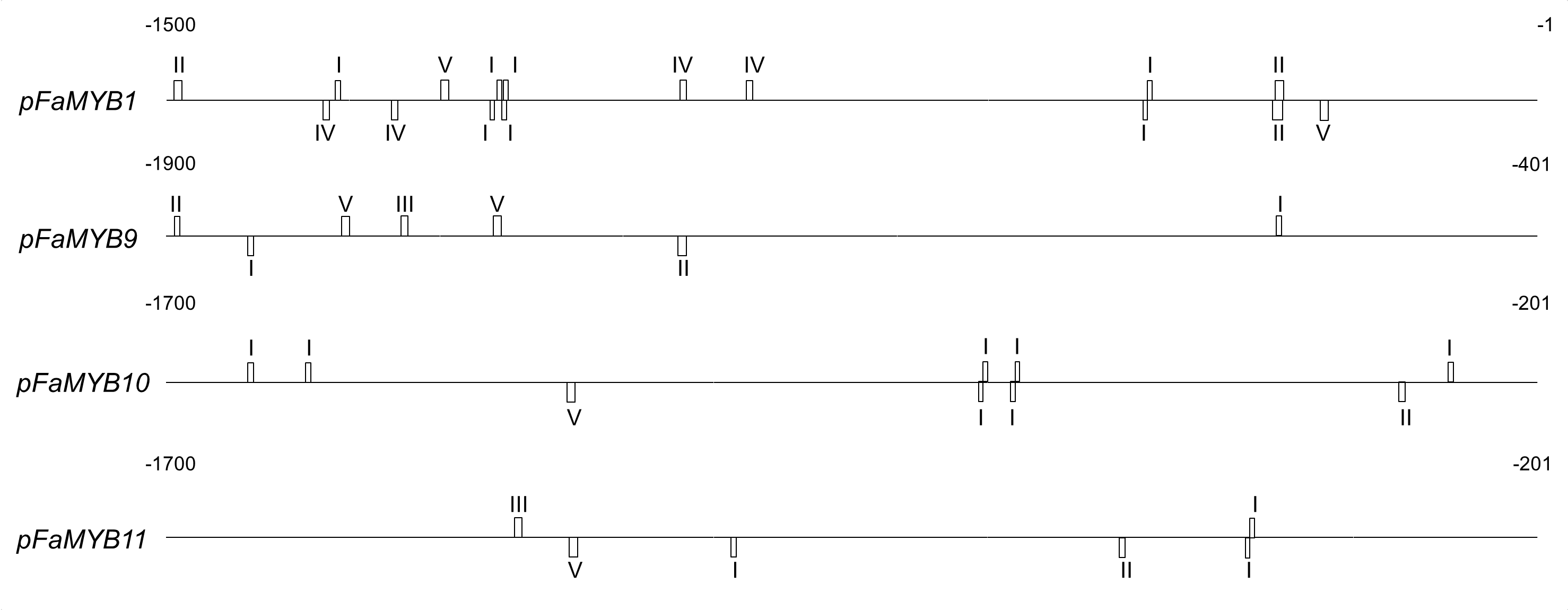

Supplement: Supplementary file 1 [file molecules-23-01433-s001.zip › Figure S1.tiff]
